# Genome-wide identification of pistil-specific genes expressed during fruit set initiation in tomato (*Solanum lycopersicum*)

**DOI:** 10.1371/journal.pone.0180003

**Published:** 2017-07-06

**Authors:** Kentaro Ezura, Kim Ji-Seong, Kazuki Mori, Yutaka Suzuki, Satoru Kuhara, Tohru Ariizumi, Hiroshi Ezura

**Affiliations:** 1 Graduate School of Life and Environmental Sciences, University of Tsukuba, Tsukuba, Ibaraki, Japan; 2 Faculty of Life and Environmental Sciences, University of Tsukuba, Tsukuba, Ibaraki, Japan; 3 Faculty of Agriculture, Kyushu University, Higashi-ku, Fukuoka, Japan; 4 Department of Computational Biology, Graduate School of Frontier Sciences, The University of Tokyo, Chiba, Japan; Chiba Daigaku, JAPAN

## Abstract

Fruit set involves the developmental transition of an unfertilized quiescent ovary in the pistil into a fruit. While fruit set is known to involve the activation of signals (including various plant hormones) in the ovary, many biological aspects of this process remain elusive. To further expand our understanding of this process, we identified genes that are specifically expressed in tomato (*Solanum lycopersicum* L.) pistils during fruit set through comprehensive RNA-seq-based transcriptome analysis using 17 different tissues including pistils at six different developmental stages. First, we identified 532 candidate genes that are preferentially expressed in the pistil based on their tissue-specific expression profiles. Next, we compared our RNA-seq data with publically available transcriptome data, further refining the candidate genes that are specifically expressed within the pistil. As a result, 108 pistil-specific genes were identified, including several transcription factor genes that function in reproductive development. We also identified genes encoding hormone-like peptides with a secretion signal and cysteine-rich residues that are conserved among some *Solanaceae* species, suggesting that peptide hormones may function as signaling molecules during fruit set initiation. This study provides important information about pistil-specific genes, which may play specific roles in regulating pistil development in relation to fruit set.

## Introduction

The pistil is a single reproductive organ that develops into a fruit after fruit set. The efficiency of fruit set is one of the most important traits that determine yield in many fruit-bearing crops such as tomato (*Solanum lycopersicum* L.). Because of its worldwide production and availability, tomato has been widely accepted as a model system for investigating fruit set. In general, fruit set is induced after successful development of the pistil upon pollination and following fertilization [1]. Through conventional molecular, genetic, and biochemical analyses of tomato, plant hormones such as auxin and gibberellic acid (GA) have been shown to play important roles in various plant developmental processes, including inducing fruit set in the pistil [1–5]. Mimicking fruit set signals by exogenous application of these hormones and mutation of the genes related to hormone signaling or metabolism induce fruit set without pollination/fertilization, a process known as parthenocarpy [6]. Furthermore, endogenous induction of auxin biosynthesis in ovules through genetic engineering is one of the most effective approaches for inducing parthenocarpy [7]. However, the key mechanisms and signals that induce fruit set in conjunction with plant hormones in the pistil remain largely unknown. To investigate this issue, it would be useful to obtain transcriptome profiles in the pistil to uncover genes regulated by signals related to fruit set.

Microarray and next generation sequencing of transcripts (RNA-Seq) are two major transcriptome profiling systems that have been widely used in molecular biology [8]. One of the benefits of transcriptome analysis is that it allows the global gene expression profiles of thousands to nearly 40,000 genes to be investigated in a single experiment. Recently, RNA-seq has become more popular than microarray analysis for obtaining transcriptome profiles and the associated quantitative data. Comparative transcriptomics by RNA-seq produces massive amounts of accurate information about differentially expressed genes between various biological events and among related individuals, providing many clues about the mechanisms underlying plant development, growth, responses to various environmental signals, and the evolution of plant species [9–15]. In studies investigating fruit development, RNA-seq-based transcriptome analyses have revealed important biological pathways and gene sets associated with fruit development and ripening [16,17–22]. However, only a limited number of transcriptome studies have targeted pistils during fruit set in tomato [20,23–25]. These studies have identified various gene sets that appear to be expressed during fruit set, such as genes related to plant hormone metabolism and sensitivity, transcription factors regulating meristem differentiation and floral organ development, and those involved in carbohydrate metabolism [20,26]. Because of their multiple effects on various aspects of plant development, it is still difficult to narrow down candidate genes or biological pathways that directly influence the induction and completion of fruit set downstream of plant hormone signaling.

Pistil comprises a mixture of heterogeneous tissues consisting of ovules, style, placenta, and pericarp (ovary wall), which often hinders the elucidation of the detailed mechanism of early fruit development due to this inherent complexity. The development of each tissue may directly influence the success of fruit set and subsequent fruit growth. After pollination, pollen enters the ovule through the style. The fertilized ovules become seeds, which provide growth signals to the entire fruit, while the rate of cell division in the ovary wall and placenta determines the final size of the fruit [1]. Recently, cell-type-specific transcriptomes of the pistil during fruit set were uncovered by two independent groups using wild tomato *S*. *pimpinellifolium* and tomato cultivar ‘Moneymaker’, providing important information about cell type-specific transcriptomes during fruit set [23,27]. In addition, several individual pistil-specific genes (PSGs) were identified, which play important roles in processes such as pollen tube extension, pollen-pistil interactions, and ovule development, highlighting the importance of PSGs in the regulation of tissue-specific development in the pistil, including two polygalacturonase genes (*PG7* and *TAPG4*) in tomato [28], one extensin-like glycoprotein gene (*PELP3*) in *Nicotiana tabacum* [29–31], one endo-1,4-β-D-glucanase gene, and one MADS box transcription factor gene (*SEEDSTOCK/AGL11*) in *Arabidopsis thaliana* [32,33]. Nonetheless, few studies have focused on the isolation of PSGs due to technical difficulties such as the small size of the tissue. Recently, anther-specific genes were identified in various species using a transcriptomic approach, which play important roles in tissue differentiation and specification [34,35]. The isolation of genes expressed in specific tissues not only provides new insights into the development of each tissue, but it also provides genetic engineering tools for molecular breeding [36]. Therefore, to extend our understanding of the molecular mechanism underlying fruit set and to generate new tools for pistil-specific regulation of fruit set-associated genes, it is important to identify PSGs that are specifically expressed during fruit set initiation.

In this study, we conducted genome-wide analysis of PSGs in tomato by RNA-seq and compared the results with publicly available data. As a result, we identified about one hundred of PSGs including genes encoding signaling-related proteins, several transcription factors, and peptide hormone-like proteins, in addition to many genes of unknown function. Further analysis of these mined genes would increase our understanding of the mechanisms underlying of pistil development and fruit set and would be useful for generating genetic engineering tools, such as tissue-specific promoters.

## Material and methods

### Plant materials, hormone treatment, and cDNA synthesis

Tomato cv ‘Micro-Tom’ was used in this study. The seeds were incubated on wet filter paper in a Petri dish at 25°C to stimulate germination, followed by growth in a cultivation room under a 16 h/8 h light/dark cycle at 25°C/22°C (day/night). Total RNA was extracted using an RNeasy Plant Mini Kit (Qiagen, USA) from 17 samples of different organs at different developmental stages: pistil and fruit samples (#1–8): pistils of 2–2.5 mm buds (#1), 3–4 mm buds (#2), 1 day before flowering (1 DBF) (#3), at anthesis (#4), 5 days after flowering (5 DAF) (#5), 5 mm ovaries of 7 days after flowering (7 DAF) (#6), mature green fruits at 33 days after flowering (MG) (#7), red fruits at 44 days after flowering (RED) (#8); stamens and other floral organ samples (#9–11): stamens of 3–4 mm buds (#9), 1 DBF (#10) and at anthesis (#11), sepals at anthesis (#12), petals at anthesis (#13), vegetative organs (#14–17 samples): 3-week-old leaves (#14), mature leaves (#15), stems (#16), and roots (#17). The total RNA was treated with DNase to remove contaminating DNA using a DNA-free RNA Kit (Zymo Research, USA). The cDNA was synthesized with 2 μg of total RNA using SuperScript VILO MasterMix (Thermo Fisher, USA) according to the manufacturer’s instructions. The cDNA libraries for RNA-seq were prepared using a TruSeq RNA Sample Prep Kit v2 (Illumina) according to manufacturer’s protocol.

### RNA-seq, processing, mapping of Illumina reads, and detection of PSGs

The 35-nt and 100-nt single-end sequencing analysis was conducted on the Illumina Genome Analyzer IIx system and Illumina HiSeq 2000, respectively. To identify the transcriptome of each tissue, “direct-mapping method” was conducted.

For the direct-mapping method, the quality of Illumina raw FASTQ data was checked by FastQC before and after trimming with Trimmomatic according to the instruction manual (S1 Fig) [37]. After trimming, only sequences with a minimum length of 20 bp were retained. The trimmed sequence data were imported into CLC Genomics Workbench ver 7.0.4 (QIAGEN, Germany) and mapped to the tomato genome SL2.50 and gene model SL2.40. Gene expression data were obtained as gene length by reads per kilobase of exon per million mapped reads (RPKM) values [38]. The data were normalized by the quantile method to reduce obscuring of variation among samples, and a logarithmic transformation part 2 subjects the normalized data after adding 1 to each values to logarithmic transformation for heat map analysis [39,40]. To narrow down the candidate genes expressed specifically in pistils, an RPKM value of 0.5 was used as the cutoff value to determine specific expression in each sample. Based on this criterion, candidate tomato PSGs with values higher than 0.5 in pistils and lower than 0.5 other tissues were identified by comparing the transcriptome data for each tissue.

### Data mining of publically available RNA-seq data

To examine the expression patterns of the identified genes in tissues other than pistils, publically available data were downloaded from transcriptome analyses of tomato from the Tomato Functional Genomics Database (http://ted.bti.cornell.edu/cgi-bin/TFGD/digital/home.cgi). Data from nine different vegetative samples from tomato cv. Heinz and wild tomato species *S*. *pimpinellifolium* were extracted and investigated to determine whether the candidate genes were expressed in these tissues.

To estimate the regions in the pistil in which the candidate genes are expressed, tissue-specific transcriptome data from the pistils of tomato wild relative *S*. *pimpinellifolium* [27] were used to identify genes with expression levels higher than RPM (reads per million mapped reads) = 2 in at least one sample. The expression levels of the top-ten genes in each tissue were then examined. To confirm the expression patterns of the candidate genes in the pistil, their expression levels were also investigated using transcriptome data from tomato cv. ‘Moneymaker’ [23]. If the expression level was higher than FPKM (Fragments Per Kilobase of exon per Million mapped fragments) 0.5 in at least one sample, it was judged to be an expressed gene. To investigate the responses of the genes to plant hormone treatment, a publically available dataset from the transcriptomes of pollinated or parthenocarpic fruit induced by hormone treatment was utilized [20]. To compare the list of differentially expressed genes with the candidate genes, unigene numbers were converted to ITAG IDs using the Unigene converter in the SGN database.

### Gene ontology analysis

ITAG IDs of the candidate PSGs were used as input with the AgriGO agricultural gene ontology (GO) analysis tool (http://bioinfo.cau.edu.cn/agriGO/analysis.php) to elucidate enriched GO terms. A false discovery rate (FDR; e-value corrected for list size) of ≤0.05 was used as the criterion to obtain enriched GO terms.

### Gene expression analysis by RT-PCR

To confirm the expression patterns of the candidate genes by RNA-seq analysis, RT-PCR was performed using cDNA samples derived from vegetative and reproductive organs, including young leaves, mature leaves, mature stems, mature roots, flower buds, and flower from 3-week-old plants. To analyze the expression patterns of the genes in ovaries or fruits before/after pollination, RT-PCR was performed using cDNA samples from tomato pistils and fruits at the corresponding developmental stages: A, pistils from 2–2.5 mm flower buds at 10 days before flowering (10 DBF); B, pistils from 3–4 mm flower buds at 7 days before flowering (7 DBF); C, pistils at 1 day before flowering (1 DBF); D, pistils at anthesis/pollination (0 DAF); E, pistils at 5 days after flowering (5 DAF); F, 5 mm ovaries at 7 days after flowering (7 DAF); G, mature green fruits at 33 days after flowering (MG); H, red fruits at 44 days after flowering (RED). Semi-quantitative reverse transcription polymerase chain reaction (RT-PCR) analysis was performed with Mastercycler ProS (Eppendorf, Germany) using an ExTaq Kit (TaKaRa Bio, Japan) and the primer sets listed in S5 Table. As an internal control for expression analysis in different organs, *SAND* expression was monitored using the primers *SAND*-F (5’- TTGCTTGGAGGAACAGACG -3’) and *SAND-*R (5’- GCAAACAGAACCCCTGAATC -3’) [41].

### Sequence analysis of genes with unknown functions

Protein sequences were downloaded from the Sol genomic network. Sequence alignments were conducted using ClustalW in DDBJ (http://clustalw.ddbj.nig.ac.jp/). Phylogenetic trees were generated using CLC Genomic Workbench. The presence of secretion signals in the small proteins was investigated using SignalP 4.1 Server (http://www.cbs.dtu.dk/services/SignalP/). The conserved domains and motifs within the identified proteins were searched using NCBI's Conserved Domain Database (CDD) (https://www.ncbi.nlm.nih.gov/Structure/cdd/wrpsb.cgi?) [42].

### Availability of RNA-seq dataset

Transcriptome data are available at the GEO database under accession number DRA005810.

## Results and discussion

### Transcriptome analysis of various tomato tissues

**Fig 1:**
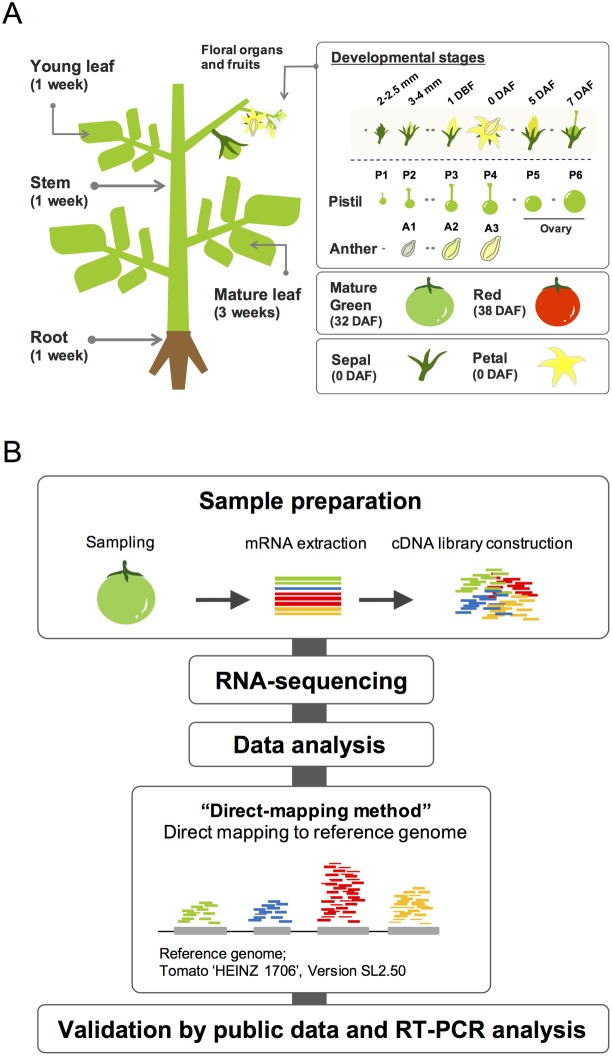
Experimental design for RNA-seq analysis. (A) The 17 samples used for transcriptome analysis. For vegetative organs, four samples were collected, including mature leaves from 3-week-old plants and young leaves, stems, and roots from 1-week-old plants. For reproductive organs and fruits, 13 samples were collected, including pistils and anthers of 2–2.5 mm buds, pistils and anthers of 3–4 mm buds, pistils at 1 day-before-flowering (1 DBF), pistils and anthers at anthesis (0 DAF), ovaries of 5-days after flowering (5 DAF), 5 mm ovaries (7 DAF), sepals and petals at anthesis, mature green fruits (MG), and red fruits (RED). (B) Work flow of transcriptome analyses. For the direct-mapping method, whole transcriptome data from short reads were obtained, which were directly mapped onto the tomato reference genome, and expressed genes were identified.

To obtain transcriptome profiles of various tomato organs in order to identify PSGs, we performed RNA-seq analysis of 17 different floral and vegetative samples at different developmental stages (Fig 1A). We initially selected six different stages for the pistil samples (P1 to P6) and three different stages for the anther samples (A1 to A3). P1 to P3 and A1 to A2 represent samples at pre-anthesis; P1 corresponds to pistils in 2–2.5 mm flower bud, P2 and A1 correspond to pistils and anthers, respectively, in 3–4 mm flower bud, and P3 and A2 correspond to those in flower buds 1 day before flowering (1 DBF), while P4 and A3 correspond to those in flower buds at anthesis (0 DAF). P5 and P6 represent samples from post-anthesis stages: P5 and P6 correspond to pistils/fruits in flowers at 5 days after flowering (5 DAF) and in 5 mm ovaries at 7 days after flowering (7 DAF) samples, respectively. In addition, we used eight samples from different tissues. We conducted 35 nt and 50 nt single reads sequencing by Illumina GAIIx and Hiseq 2000, respectively (S1 Table). We used the “direct-mapping method” to identify sets of PSGs (Fig 1B). In the direct-mapping method, whole sequenced short reads were directly mapped onto the tomato reference genome.

The direct-mapping method is a common approach for transcriptome analysis in which sequence reads are mapped onto the reference genome of a target organism [8,38]. Our RNA-seq generated different amounts of raw data ranging from 8.32 to 39.42 million reads. After quality checking and trimming of low quality reads and adapter sequences, we obtained 7.28 to 35.23 million clean reads for mapping (S1 Table). We analyzed the reads using CLC Genomic Workbench ver. 7.0.4, a user-friendly mapping tool; 82.3% to 90.5% of the clean reads from each sample were mapped to the tomato genome SL2.40 [43] (S1 Table). An RPKM cutoff value of 0.5 was utilized to declare a locus expressed, resulting in an average of approximately 25,000 genes above the expression threshold in 17 samples (S2A Fig).

**Fig 2:**
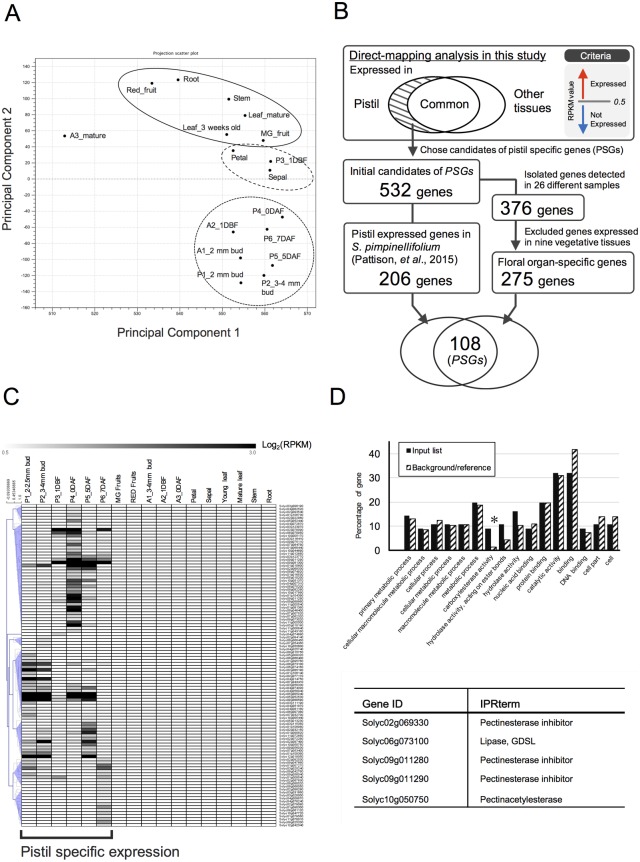
Identification of genes preferentially expressed in pistils based on the direct-mapping method. (A) PCA analysis of the RNA-seq data. (B) Identification and validation of the expression of 532 candidate genes with RPKM values greater than 0.5 in at least one pistil sample and less than 0.5 in the other tissue samples using publically available data. Out of 532 genes, 206 were found to be expressed in the transcriptome produced by Pattison et al. (2015) [27]. On the other hand, the expression of 376 genes was detected in at least one sample from many different tissues and conditions, and 275 of these showed RPKM values less than 1 in nine vegetative samples (Floral organ specific genes). Finally, by comparing the two gene sets, 108 genes were found in both sets, identified as pistil-specific genes (PSGs). (C) Heatmap of the expression of 108 genes in 17 different samples. Normalized Log2-transformed expression data were visualized by constructing a heatmap using MeV software. Hierarchical clustering by Pearson correlation was conducted. (D) Gene ontology analysis was performed using AgriGO (http://bioinfo.cau.edu.cn/agriGO/). Only one category, carboxylesterase activity (GO:0004091), was significantly (FDR<0.05) represented in the gene set, while 56 genes were not annotated and were not assigned to GO terms. bottom table; Gene ID and functional annotation described in the SGN database.

Before isolating PSGs, we examined the quality of our transcriptome data, as we used only one replicate per sample. Initially, to characterize the transcriptome data, we conducted principal component analysis (PCA) with CLC Genomic Workbench ver. 7.0.4. Component 1 explained 91% of the variation, while component 2 explained 2% of the variation, indicating that the two components together explained 93% of the variation of the 17 original variables. Samples from vegetative and reproductive organs were separated into two groups, with samples such as petals and sepals (which are components of reproductive organ but are composed of vegetative cells) located in the middle of the two groups (Fig 2A), indicating specialized transcriptomes. We investigated the expression patterns of the homologs that had already been identified as tissue-specific genes, such as YABBY transcription factor genes. In Arabidopsis, two YABBY transcription factors, *CRABS CLAW* (*CRC*) and *INNER NO OUTER* (*INO*), show pistil-specific expression and are involved in the pistil and early fruit development [44–46]. Thus, we expected the expression patterns of their homologs in tomato to show pistil-specific expression, and we therefore examined this possibility. Three of nine YABBY transcription factor genes found in the tomato genome, *SlCRCa* (*Solyc01g0101240*), *SlCRCb* (*Solyc05g012050*), and *SlINO* (*Solyc05g005240*), were specifically expressed in flower buds and flower at the anthesis stage, which is consistent with the results obtained in a previous study [47] (S2B Fig). *SlCRCa* was expressed in the early stage of pistil development, and *SlCRCb* and *SlINO* were expressed through all stages of pistil development, while they were barely expressed in the other tissues (S2B Fig). These data support the quality of the transcriptome dataset. Next, according to RPKM values, we narrowed down the list of genes to those with RPKM values greater than 0.5 in at least one pistil sample and less than 0.5 in the other tissues, resulting in the identification of 532 of the initial candidate PSGs obtained by the direct-mapping method (Fig 2B).

### Validation of the expression specificity of the candidate genes using publically available datasets

To reconfirm the tissue-specific expression of the 532 candidate genes, we performed comparative analyses between our transcriptome dataset and two publicly available transcriptome datasets (Experiment 1 and Experiment 2) from 26 samples, including vegetative tissues and floral tissues derived from tomato cv. Heinz and wild relative *S*. *pimpinellifolium* (strain. LA1589) available in the Tomato Functional genomics database (http://ted.bti.cornell.edu/); Experiment 1 (Exp1; Tomato Genome Consortium, 2012), Experiment 2 (Exp2; accession no. PRJNA179156). As a result, 376 of the 532 candidate genes were detected in at least one of the 26 samples from the public data, suggesting that these genes are most likely expressed in tomato plants. We investigated the expression levels of the 376 genes in nine different vegetative samples. We then excluded genes whose RPKM values were >1 in any of nine vegetative samples and identified 275 genes as “Floral organ-specific genes” (Fig 2B).

Alternatively, to obtain information about the cell types in which the candidate genes are expressed, we investigated their expression patterns in cell-type-specific transcriptome data from pistils of wild tomato (*S*. *pimpinellifolium*) [27]. We then selected genes expressed in pistils based on the criterion used by Pattison et al. [27]; genes with RPM values > 2 in at least one sample were chosen. In total, 206 genes were defined as “Pistil expressed genes”; their expression was evident in the pistil, especially after anthesis, while the other 326 genes excluded by this step may not be expressed in the pistil or may be expressed only at the earlier stages than 1 DBF (Fig 2B).

**Fig 3:**
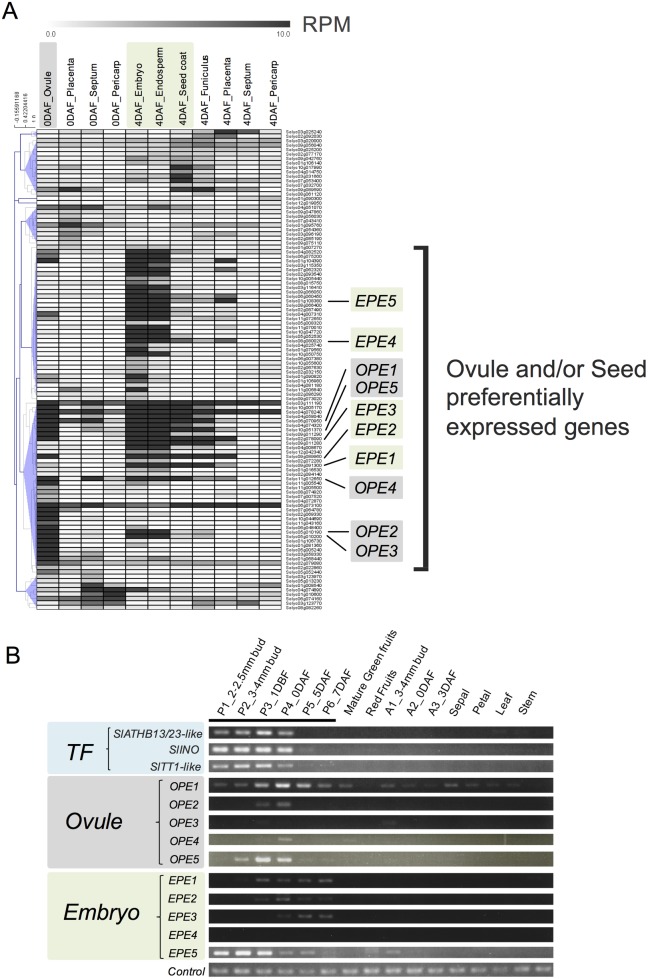
Characterization and validation of the 108 genes. (A) Many of the 108 PSGs were predominantly expressed in ovules and/or seeds in the pistil. Reads per million (RPM) values of PSGs in *S*. *pimpinellifolium* were visualized by constructing a heatmap using MeV software. Hierarchical clustering by Pearson correlation was conducted. The transcriptome data were obtained from [27]. *OPE*; ovule preferentially expressed genes, *EPE*; embryo preferentially expressed genes. (B) Validation of the expression of ovule preferentially expressed (*OPE*) genes, embryo preferentially expressed (*EPE*) genes, and several transcription factor genes by RT-PCR. Most of the genes were specifically expressed in the pistil. Three pistil-specific transcription factor genes, *SlATHB13/23-like* (*Solyc01g010600*), *SlINO* (*Solyc05g005240*), and *SlTT1* (*Solyc10g051370*) showed pistil-specific expression before anthesis. Bottom one represents the expression of the internal control gene SAND [41].

We compared “Floral organ-specific genes” and “Pistil expressed genes” and selected redundant genes, ultimately identifying 108 genes as PSGs by the direct-mapping method (Fig 2B and 2C, Table 1 and S2 Table). Among these, 56 genes had not been characterized. Public transcriptome data analysis provided information about both the organs and cell types in which the 108 *PSG*s were expressed. Using cell-type-specific transcriptome dataset from pistils of wild relative *S*. *pimpinellifolium* [27], we obtained spatial information about the expression of *PSGs* within the pistil (S3 Table). Hierarchical heat mapping clearly showed their cell-type-specific expression profiles (Fig 3A). Remarkably, roughly two-thirds of the genes appeared to show highly tissue-specific expression in the ovule and/or seed tissues (embryo, endosperm, seed coat). While many genes were preferentially expressed in the ovule and the seed tissues except seed coat, several genes were preferentially expressed in the pericarp at anthesis, in the placenta, and in the seed coat after pollination (Fig 3A). For example, five genes were preferentially expressed in the pericarp before pollination: genes encoding cinnamoyl CoA reductase-like protein (*Solyc01g008540*), Unknown Protein (*Solyc04g074890*), homeobox-leucine zipper-like protein (*Solyc01g010600*), B3 domain-containing protein Os03g0212300–like protein (*Solyc06g074160*), and Unknown Protein (*Solyc03g123770*). Furthermore, the gene encoding cytokinin oxidase/dehydrogenase 8 (*SlCKX8*, *Solyc10g017990*), TNFR/CD27/30/40/95 cysteine-rich region (*Solyc04g014750*), Unknown Protein (*Solyc03g031660*), Unknown Protein (*Solyc07g053400*), and Ramosa1 C2H2 zinc-finger transcription factor (*Solyc09g089590*) were preferentially expressed in the seed coat. *Solyc09g089590* encodes one of two homologous proteins of Arabidopsis SUPERMAN (SUP), which regulates auxin biosynthesis [48]. In addition, the expression of 63 out of 108 genes was detected also in the recently published ovary transcriptome dataset derived from cultivated tomato ‘Moneymaker’ [23], in which RNA-seq analyses against ovule and ovary wall tissue were conducted; their average expression levels were over FPKM of 0.5 [23] (S4 Table). The 55 other genes were not detected in that dataset, indicating that these 55 genes were barely expressed in cultivated tomato or were only expressed in other type of tissues such as the placenta and septum, which were excluded from their experiment.

**Table 1:** List of 108 pistil-specific genes (*PSGs*) identified by the direct-mapping-based method.

#	ITAG ID	Description in ITAG2.40	Homologue in Arabidopsis	length (aa)	Identities (%)		
1	Solyc01g007270	Cytokinin riboside 5&apos;-monophosphate phosphoribohydrolase LOG (AHRD V1 **—LOG_ORYSJ)	AT5G06300	217	56/68	82	
2	Solyc01g008540	Cinnamoyl CoA reductase-like protein (AHRD V1 ***- B9HNY0_POPTR); Interpro domain(s) IPR016040 NAD(P)-binding domain	AT5G19440	326	223/315	71	NAD(P)-binding Rossmann-fold superfamily protei
3	Solyc01g010600	Homeobox-leucine zipper-like protein (AHRD V1 *-*- Q3HRT1_PICGL); contains In contains terpro domain(s) IPR001356 Homeobox	AT1G69780	294	150/300	50	ATHB13
4	Solyc01g016530	Unknown Protein (AHRD V1); contains Interpro domain(s) IPR008507 Protein of unknown function DUF789	AT1G73210	314	32/69	46	Protein of unknown function (DUF789)
5	Solyc01g068440	Os06g0207500 protein (Fragment) (AHRD V1 ***- Q0DDQ9_ORYSJ); contains Interpro domain(s) IPR004253 Protein of unknown function DUF231, plant	AT2G42570	367	166/341	49	TBL39 (TRICHOME BIREFRINGENCE-LIKE 39 )
6	Solyc01g079560	B3 domain-containing protein Os11g0197600 (AHRD V1 ***- Y1176_ORYSJ); contains Interpro domain(s) IPR003340 Transcriptional factor B3	AT3G18990	341	30/92	33	VRN1, REM39
7	Solyc01g081360	Unknown Protein (AHRD V1)	-	-	-	-	
8	Solyc01g090300	Ethylene responsive transcription factor 1b (AHRD V1 *-*- C0J9I8_9ROSA); contains Interpro domain(s) IPR001471 Pathogenesis-related transcriptional factor and ERF, DNA-binding	AT2G44840	226	69/107	64	ATERF13, EREBP, ERF13
9	Solyc01g090820	Expansin B1 (AHRD V1 ***- C8CC40_RAPSA); contains Interpro domain(s) IPR007112 Expansin 45, endoglucanase-like	AT1G65680	273	119/249	48	ATEXPB2, EXPB2, ATHEXP BETA 1.4
10	Solyc01g095760	UDP-glucosyltransferase (AHRD V1 ***- Q8LKG3_STERE); contains Interpro domain(s) IPR002213 UDP-glucuronosyl/UDP-glucosyltransferase	AT5G49690	460	164/471	35	UDP-Glycosyltransferase superfamily protein
11	Solyc01g104390	Blue copper protein (AHRD V1 **—B6TT37_MAIZE); contains Interpro domain(s) IPR003245 Plastocyanin-like	AT1G17800	129	49/116	42	ARPN
12	Solyc01g106140	F-box protein family-like (AHRD V1 *-*- Q6ZCS3_ORYSJ); contains Interpro domain(s) IPR005174 Protein of unknown function DUF295	AT3G25750	348	41/162	25	F-box family protein with a domain of unknown function (DUF295)
13	Solyc01g106730	MADS box transcription factor 1 (AHRD V1 *-*- D9IFM1_ONCHC); contains Interpro domain(s) IPR002100 Transcription factor, MADS-box	AT5G60440	299	95/160	59	AGL62
14	Solyc01g106980	Endo-1 4-beta-xylanase (AHRD V1 *—B6SW51_MAIZE); contains Interpro domain(s) IPR013781 Glycoside hydrolase, subgroup, catalytic core	AT4G33840	576	276/545	51	Glycosyl hydrolase family 10 protein
15	Solyc01g108380	Protease inhibitor protein (AHRD V1 -**- B3FNP9_HEVBR); contains Interpro domain(s) IPR000864 Proteinase inhibitor I13, potato inhibitor I	AT2G38900	88	27/61	44	Serine protease inhibitor, potato inhibitor I-type family protein
16	Solyc02g022860	FAD-binding domain-containing protein (AHRD V1 **—D7MFI0_ARALY); contains Interpro domain(s) IPR006094 FAD linked oxidase, N-terminal	AT4G20820	532	243/532	46	FAD-binding Berberine family protein
17	Solyc02g032150	Unknown Protein (AHRD V1)	-	-	-	-	-
18	Solyc02g067630	Polygalacturonase 1 (AHRD V1 ***- O22311_SOLLC); contains Interpro domain(s) IPR000408 Regulator of chromosome condensation, RCC1 IPR000743 Glycoside hydrolase, family 28	AT2G43860	384	232/389	60	Pectin lyase-like superfamily protein
19	Solyc02g069330	Unknown Protein (AHRD V1); contains Interpro domain(s) IPR006501 Pectinesterase inhibitor	AT5G64620	180	26/80	33	C/VIF2, ATC/VIF2
20	Solyc02g072280	Subtilisin-like protease (AHRD V1 **—Q9LWA3_SOLLC); contains Interpro domain(s) IPR015500 Peptidase S8, subtilisin-related	AT5G67360	757	335/761	44	ARA12
21	Solyc02g077170	X1 (Fragment) (AHRD V1 *—Q7FSP8_MAIZE); contains Interpro domain(s) IPR005379 Region of unknown function XH	AT1G15910	634	96/259	37	XH/XS domain-containing protein
22	Solyc02g078090	Unknown Protein (AHRD V1)	-	-	-	-	-
23	Solyc02g079080	F-box family protein (AHRD V1 ***- B9GFH4_POPTR); contains Interpro domain(s) IPR001810 Cyclin-like F-box	AT5G02930	469	108/440	25	F-box/RNI-like superfamily protein
24	Solyc02g084140	Unknown Protein (AHRD V1)	-	-	-	-	-
25	Solyc02g085190	GATA transcription factor 19 (AHRD V1 *-** B6TS85_MAIZE); contains Interpro domain(s) IPR000679 Zinc finger, GATA-type	AT3G50870	295	127/292	43	MNP, HAN, GATA18
26	Solyc02g086290	Receptor serine/threonine kinase (AHRD V1 ***- Q9FF31_ARATH)	AT1G66940	332	76/267	28	protein kinase-related
27	Solyc02g087490	Prolyl 4-hydroxylase alpha subunit-like protein (AHRD V1 ***- Q9LSI6_ARATH); contains Interpro domain(s) IPR006620 Prolyl 4-hydroxylase, alpha subunit	AT3G28490	288	176/265	66	Oxoglutarate/iron-dependent oxygenase
28	Solyc02g092030	Cbs domain containing protein expressed (Fragment) (AHRD V1 *—A6N095_ORYSI); contains Interpro domain(s) IPR002550 Protein of unknown function DUF21	AT2G14520	423	283/423	67	CBS domain-containing protein with a domain of unknown function (DUF21)
29	Solyc02g093540	Cytochrome P450	AT3G50660	513	193/470	41	DWF4, CYP90B1, CLM, SNP2, SAV1, PSC1
30	Solyc03g020000	Pentatricopeptide repeat-containing protein (AHRD V1 *-*- D7L041_ARALY); contains Interpro domain(s) IPR002885 Pentatricopeptide repeat	AT2G22410	681	181/487	37	SLO1
31	Solyc03g025240	Multidrug resistance protein mdtK (AHRD V1 *—MDTK_YERP3); contains Interpro domain(s) IPR002528 Multi antimicrobial extrusion protein MatE	AT4G25640	514	273/398	69	DTX35
32	Solyc03g031660	Unknown Protein (AHRD V1)	-	-	-	-	-
33	Solyc03g058330	Unknown Protein (AHRD V1)	AT5G06760	158	57/144	40	LEA4-5
34	Solyc03g096190	Receptor like kinase, RLK	AT3G47570	1010	441/1003	43	Leucine-rich repeat protein kinase family protein
35	Solyc03g111190	Auxin-independent growth promoter-like protein (AHRD V1 ***- Q9FMW3_ARATH); contains Interpro domain(s) IPR004348 Protein of unknown function DUF246, plant	AT5G63390	559	343/557	62	O-fucosyltransferase family protein
36	Solyc03g115350	Expansin 2 (AHRD V1 ***- C0KLG9_PYRPY); contains Interpro domain(s) IPR002963 Expansin	AT5G39280	259	146/223	65	ATEXPA23, ATEXP23, ATHEXP ALPHA 1.17
37	Solyc03g116410	Zinc finger CCCH domain-containing protein 39 (AHRD V1 ***- C3H39_ARATH); contains Interpro domain(s) IPR000571 Zinc finger, CCCH-type	AT3G19360	386	54/199	27	Zinc finger (CCCH-type) family protein
38	Solyc03g123770	Unknown Protein (AHRD V1)	-	-	-	-	-
39	Solyc03g123970	Lipid-binding serum glycoprotein family protein (AHRD V1 *-*- D7LAX8_ARALY)	AT3G20270	722	26/51	51	lipid-binding serum glycoprotein family
40	Solyc04g007310	Thaumatin-like protein (AHRD V1 ***- C1K3P2_PYRPY); contains Interpro domain(s) IPR001938 Thaumatin, pathogenesis-related	AT4G38670	253	108/252	43	Pathogenesis-related thaumatin superfamily protein
41	Solyc04g008670	Gibberellin 2-beta-dioxygenase 7 (AHRD V1 **** B6SZM8_MAIZE); contains Interpro domain(s) IPR005123 Oxoglutarate and iron-dependent oxygenase	AT4G21200	336	166/302	55	ATGA2OX8, GA2OX8
42	Solyc04g014750	TNFR/CD27/30/40/95 cysteine-rich region (AHRD V1 ***- Q2HT38_MEDTR)	AT1G12064	109	34/73	47	Unkown protein
43	Solyc04g025740	Homeobox-leucine zipper protein ROC3 (AHRD V1 ***- ROC3_ORYSJ); contains Interpro domain(s) IPR001356 Homeobox	AT1G73360	722	52/125	42	HDG11, EDT1, ATHDG11
44	Solyc04g051070	Unknown Protein (AHRD V1)	-	-	-	-	-
45	Solyc04g058040	Laccase (AHRD V1 ***- Q9AUI3_PINTA); contains Interpro domain(s) IPR011707 Multicopper oxidase, type 3	AT5G09360	569	82/212	39	LAC14
46	Solyc04g072870	Beta-D-xylosidase (AHRD V1 **** Q8W011_HORVU); contains Interpro domain(s) IPR001764 Glycoside hydrolase, family 3, N-terminal	AT1G78060	767	445/756	59	Glycosyl hydrolase family protein
47	Solyc04g074320	Zinc finger protein (AHRD V1 *—D7KHP2_ARALY); contains Interpro domain(s) IPR007087 Zinc finger, C2H2-type	AT1G34790	303	143/200	72	TT1, WIP1
48	Solyc04g074890	Unknown Protein (AHRD V1)	-	-	-	-	-
49	Solyc04g078240	Natural resistance associated macrophage protein (AHRD V1 *—B3W4E1_BRAJU); contains Interpro domain(s) IPR001046 Natural resistance-associated macrophage protein	AT1G47240	530	73/95	77	NRAMP2, ATNRAMP2
50	Solyc04g081180	Unknown Protein (AHRD V1)	-	-	-	-	-
51	Solyc04g082520	Ring zinc finger protein (Fragment) (AHRD V1 *—A6MH00_LILLO); contains Interpro domain(s) IPR008166 Protein of unknown function DUF23	AT4G37420	588	233/500	47	Domain of unknown function (DUF23)
52	Solyc05g005240	YABBY-like transcription factor CRABS CLAW-like protein (AHRD V1 **-* Q6SRZ7_ANTMA); contains Interpro domain(s) IPR006780 YABBY protein	AT1G23420	231	100/184	54	INO
53	Solyc05g008320	Fasciclin-like arabinogalactan protein (AHRD V1 ***- B9N201_POPTR); contains Interpro domain(s) IPR000782 FAS1 domain	AT5G40940	424	114/328	35	FLA20
54	Solyc05g010190	Unknown Protein (AHRD V1)	AT3G42565	119	48/121	40	ECA1 gametogenesis related family protein
55	Solyc05g010200	Unknown Protein (AHRD V1)	-	-	-	-	-
56	Solyc05g013230	Unknown Protein (AHRD V1)	AT3G23880	364	21/57	37	F-box and associated interaction domains-containing protein
57	Solyc05g052440	Os03g0291800 protein (Fragment) (AHRD V1 **—Q0DSS4_ORYSJ); contains Interpro domain(s) IPR004253 Protein of unknown function DUF231, plant	AT2G40320	425	279/411	68	TBL33
58	Solyc05g052530	Endoglucanase 1 (AHRD V1 ***- B6U0J0_MAIZE); contains Interpro domain(s) IPR001701 Glycoside hydrolase, family 9	AT2G44550	490	292/476	56	ATGH9B10
59	Solyc06g007380	Os08g0119500 protein (Fragment) (AHRD V1 *-*- Q0J8C9_ORYSJ)	AT5G01710	513	258/510	51	methyltransferases
60	Solyc06g048400	Unknown Protein (AHRD V1); contains Interpro domain(s) IPR008502 Protein of unknown function DUF784, Arabidopsis thaliana	AT3G30387	115	34/97	35	Protein of unknown function (DUF784)
61	Solyc06g060450	Transmembrane emp24 domain-containing protein 10 (AHRD V1 ***- B6SSF8_MAIZE); contains Interpro domain(s) IPR000348 emp24/gp25L/p24	AT1G2190	216	108/210	51	emp24/gp25L/p24 family/GOLD family protein
62	Solyc06g070950	ATP-binding cassette (ABC) transporter 17 (AHRD V1 ***- Q4H493_RAT); contains Interpro domain(s) IPR003439 ABC transporter-like	AT3G47780	935	503/937	54	ATATH6, ATH6
63	Solyc06g073100	GDSL esterase/lipase At3g27950 (AHRD V1 ***- GDL54_ARATH); contains Interpro domain(s) IPR001087 Lipase, GDSL	AT3G27950	361	197/375	53	GDSL-like Lipase/Acylhydrolase superfamily protein
64	Solyc06g074160	B3 domain-containing protein Os03g0212300 (AHRD V1 ***- Y3123_ORYSJ); contains Interpro domain(s) IPR003340 Transcriptional factor B3	AT3G06160	374	38/131	29	AP2/B3-like transcriptional factor family protein
65	Solyc06g075200	Unknown Protein (AHRD V1)	AT5G37474	80	28/83	34	Putative membrane lipoprotein
66	Solyc07g007520	Unknown Protein (AHRD V1)	-	-	-	-	-
67	Solyc07g032700	Unknown Protein (AHRD V1)	-	-	-	-	-
68	Solyc07g043410	UDP-glucosyltransferase family 1 protein (AHRD V1 **** C6KI43_CITSI); contains Interpro domain(s) IPR002213 UDP-glucuronosyl/UDP-glucosyltransferase	AT2G15480	484	166/487	34	UGT73B5
69	Solyc07g053400	Unknown Protein (AHRD V1)	-	-	-	-	-
70	Solyc07g054360	Unknown Protein (AHRD V1)	-	-	-	-	-
71	Solyc07g062320	Unknown Protein (AHRD V1)	-	-	-	-	-
72	Solyc07g064780	Unknown Protein (AHRD V1)	-	-	-	-	-
73	Solyc08g015750	F-box family protein (AHRD V1 ***- B9I6K2_POPTR); contains Interpro domain(s) IPR001810 Cyclin-like F-box	AT5G02920	469	58/200	31	F-box/RNI-like superfamily protein
74	Solyc08g061120	Unknown Protein (AHRD V1)	-	-	-	-	-
75	Solyc08g066400	Protein kinase (Fragment) (AHRD V1 *-*- A2Q5N5_MEDTR)	AT2G25760	676	217/333	65	Protein kinase family protein
76	Solyc08g074920	Aspartic proteinase nepenthesin I (AHRD V1 **—A9ZMF9_NEPAL); contains Interpro domain(s) IPR001461 Peptidase A1	AT5G33340	437	206/437	47	CDR1
77	Solyc08g080020	Serine protease inhibitor potato inhibitor I-type family protein (AHRD V1 ***- D7LT19_ARALY); contains Interpro domain(s) IPR000864 Proteinase inhibitor I13, potato inhibitor I	AT3G46860	85	32/86	37	Serine protease inhibitor, potato inhibitor I-type family protein
78	Solyc08g082260	Integrin-linked kinase-associated serine/threonine phosphatase 2C (AHRD V1 **** ILKAP_RAT); contains Interpro domain(s) IPR015655 Protein phosphatase 2C	AT2G29380	362	134/298	45	HAI3
79	Solyc09g011280	Unknown Protein (AHRD V1); contains Interpro domain(s) IPR006501 Pectinesterase inhibitor	AT3G17220	173	31/131	24	ATPMEI2
80	Solyc09g011290	Invertase inhibitor homolog (AHRD V1 ***- O49603_ARATH); contains Interpro domain(s) IPR006501 Pectinesterase inhibitor	AT5G64620	180	52/173	30	C/VIF2, ATC/VIF2
81	Solyc09g025200	Ribosomal protein L18 (AHRD V1 *-*- B7FMF5_MEDTR); contains Interpro domain(s) IPR000039 Ribosomal protein L18e	AT3G05590	187	31/50	62	RPL18
82	Solyc09g042760	ZIP4/SPO22 (AHRD V1 **—A5Y6I6_ARATH); contains Interpro domain(s) IPR013940 Meiosis specific protein SPO22	AT5G48390	936	527/936	56	ATZIP4
83	Solyc09g047860	HAT family dimerisation domain containing protein (AHRD V1 *-*- Q2R1C3_ORYSJ); contains Interpro domain(s) IPR008906 HAT dimerisation	AT5G33406	509	52/173	30	hAT dimerisation domain-containing protein / transposase-related
84	Solyc09g056030	Unknown Protein (AHRD V1)	AT4G12570	873	17/44	39	UPL5
85	Solyc09g056040	Ubiquitin-protein ligase 1 (AHRD V1 ***- Q5CHN2_CRYHO); contains Interpro domain(s) IPR000569 HECT	AT4G12570	873	153/413	37	UPL5
86	Solyc09g066050	Homeodomain-containing transcription factor FWA (AHRD V1 **-* B5BQ02_ARASU); contains Interpro domain(s) IPR002913 Lipid-binding START	AT1G73360	722	211/587	36	HDG11, EDT1, ATHDG11
87	Solyc09g073020	Unknown Protein (AHRD V1)	-	-	-	-	-
88	Solyc09g075110	Unknown Protein (AHRD V1)	-	-	-	-	-
89	Solyc09g089590	Ramosa1 C2H2 zinc-finger transcription factor (AHRD V1 *-*- D0UTY8_ZEAMM); contains Interpro domain(s) IPR007087 Zinc finger, C2H2-type	AT3G23130	204	78/192	78	SUP, FON1, FLO10
90	Solyc09g089960	Unknown Protein (AHRD V1)	-	-	-	-	
91	Solyc09g091300	Self-incompatibility protein (Fragment) (AHRD V1 -**- C8C1B5_9MAGN); contains Interpro domain(s) IPR010264 Plant self-incompatibility S1	AT3G26880	161	35/135	33	Plant self-incompatibility protein S1 family
92	Solyc10g005170	Purine permease (AHRD V1 *—* B6TET5_MAIZE); contains Interpro domain(s) IPR004853 Protein of unknown function DUF250	AT1G30840	382	208/330	63	ATPUP4, PUP4
93	Solyc10g005440	Serine/threonine-protein kinase receptor (AHRD V1 **** B6U2B7_MAIZE); contains Interpro domain(s) IPR002290 Serine/threonine protein kinase	AT4G21390	849	440/858	51	B120, S-locus lectin protein kinase family protein
94	Solyc10g017990	Cytokinin oxidase/dehydrogenase 2 (AHRD V1 *-** C0LPA7_SOLTU); contains Interpro domain(s) IPR015345 Cytokinin dehydrogenase 1, FAD and cytokinin binding	AT2G41510	575	214/525	41	ATCKX1, CKX1
95	Solyc10g044690	Annexin (AHRD V1 ***- D2D2Z9_GOSHI); contains Interpro domain(s) IPR009118 Annexin, type plant	AT5G12380	316	173/316	55	ANNAT8
96	Solyc10g047720	Unknown Protein (AHRD V1)	AT5G26805	156	44/163	27	unknown protein
97	Solyc10g050750	Pectinacetylesterase like protein (Fragment) (AHRD V1 *—Q56WP8_ARATH); contains Interpro domain(s) IPR004963 Pectinacetylesterase	AT4G19420	397	234/381	61	Pectinacetylesterase family protein
98	Solyc10g051370	LRR receptor-like serine/threonine-protein kinase, RLP	AT2G16250	915	105/198	53	Leucine-rich repeat protein kinase family protein
99	Solyc10g055600	S-phase kinase-associated protein 1A (AHRD V1 **—B2VUU5_PYRTR); contains Interpro domain(s) IPR001232 SKP1 component	AT4G34210	152	38/47	81	ASK11, SK11
100	Solyc11g005500	ECA1 protein (AHRD V1 *-*- Q53JF8_ORYSJ); contains Interpro domain(s) IPR010701 Protein of unknown function DUF1278	AT1G76750	158	63/124	51	EC1.1
101	Solyc11g005540	ECA1 protein (AHRD V1 *-*- Q53JF8_ORYSJ); contains Interpro domain(s) IPR010701 Protein of unknown function DUF1278	AT2G21750	125	61/130	47	EC1.3
102	Solyc11g006840	Unknown Protein (AHRD V1)	-	-	-	-	-
103	Solyc11g012650	TPD1 (AHRD V1 *-*- Q6TLJ2_ARATH)	AT1G32583	179	66/112	59	TPD1-like
104	Solyc11g043160	Endo-1 4-beta-xylanase (AHRD V1 ***- B6SW51_MAIZE); contains Interpro domain(s) IPR013781 Glycoside hydrolase, subgroup, catalytic core	AT4G33840	576	217/545	40	Glycosyl hydrolase family 10 protein
105	Solyc11g070010	F8A5.6 protein (AHRD V1 **—Q9ZP57_ARATH)	AT1G60500	669	117/391	30	DRP4C
106	Solyc11g072650	Trans-2-enoyl CoA reductase (AHRD V1 **—C5MRG3_9ROSI); contains Interpro domain(s) IPR002085 Alcohol dehydrogenase superfamily, zinc-containing	AT3G45770	375	215/335	64	Polyketide synthase, enoylreductase
107	Solyc12g019050	Exostosin-like (AHRD V1 ***- A4Q7M8_MEDTR); contains Interpro domain(s) IPR004263 Exostosin-like	AT3G42180	470	203/349	57	Exostosin family protein
108	Solyc12g042340	Genomic DNA chromosome 5 P1 clone MAC9 (AHRD V1 ***- Q9FLS4_ARATH)	AT5G61865	417	136/368	35	unknown protein

### Validation of gene expression patterns by RT-PCR

We then verified the expression patterns of the PSGs by RT-PCR analysis. Since many of these genes were highly expressed in the ovule and/or seed, especially the embryo (Fig 3A), we initially focused on genes specifically expressed in these tissues. Among the 108 PSG candidates, the top-five PSGs highly expressed in 0 DAF ovules were designated Ovule Preferentially Expressed genes 1–10 (*OPE1–5*) (S5 Table). We verified the tissue-specific expression of five of these genes by RT-PCR analysis (Fig 3B). *OPE1* was preferentially but not exclusively expressed in the pistil at anthesis, *OPE2* and *OPE5* were specifically expressed in the pistil at both 1 DBF and 0 DAF, and the expression of *OPE3* in the pistil was not detected in this experiment. *OPE4* was expressed in the pistil at 0 DAF and mature green fruits. We also designated the top-five PSGs that were highly expressed in 4 DAF embryos as Embryo Preferentially Expressed genes 1–5 (*EPE1–5*) (S5 Table). *EPE1*, encoding a self-incompatibility protein-like protein according to SGN, might function in pollen-pistil interactions, while most of the *EPEs* had not been functionally characterized or annotated in previous studies. Like the *OPE*s, we investigated the expression of the five *EPEs* (*EPE1–5*) by RT-PCR to validate their tissue-specific expression patterns. Three genes, *EPE1*-*EPE3*, were specifically expressed in the pistil and EPE5 was preferentially expressed in the pistil especially before anthesis, although we failed to detect the expression of *EPE4* in our RT-PCR analysis (Fig 3B). *EPE1* was specifically expressed in the pistil throughout pistil/fruit development but was not expressed in mature red fruits. *EPE3* was also specifically expressed in the pistil, but only after anthesis. *EPE2* was expressed exclusively during fruit set initiation between 1 DBF and 0 DAF (Fig 3B). In summary, three *OPE*s and four *EPE*s were specifically expressed in pistils, confirming their tissue-specific expression in the pistil (Fig 3B). Therefore, we confirmed the tissue-specific expression of *PSGs* in the pistil. These results indicate that the direct-mapping method also successfully identified true PSGs.

### GO analysis using AgriGO

To elucidate the enriched functional categories of the 108 identified PSGs, we performed GO analysis using AgriGO. A false discovery rate (FDR; e-value corrected for list size) of < 0.05 was used as the criterion to obtain enriched GO terms. Consequently, only one category, Carboxylesterase activity (GO:0004091), showed significant abundance (p-value = 0.0017, FDR = 0.037) (Fig 2D). This category includes five genes (listed in Fig 2D), three of which (*Solyc02g069330*, *Solyc09g011280*, and *Solyc09g011290*) were assigned to the sub-term “Pectinesterase inhibitor”. Even though *Solyc09g011290* was classified as a “Pectinesterase inhibitor”, it was labeled as an “invertase inhibitor homolog” in the SGN database and has higher sequence homology with the invertase inhibitor group that includes *invertase inhibitor 1* (*INVINH1*, *Solyc12g099200*), which specifically regulates cell wall invertase activity in early developing fruits [49].

Pectin, a major component of the primary cell walls of higher plants, is methyl-esterified by pectin methyltransferase (PMT) before its transport to the cell wall following its biosynthesis in Golgi bodies [50,51], whereas pectin methylesterase (PME) catalyzes the removal of methyl esters from pectin [52–54]. The removal of methyl group from pectin allows carboxyl groups to form Ca^2+^- and Mg^2+^-mediated linkages, leading to the hardening of pectin [55,56]. In addition, pectin methylesterase inhibitors (PMEIs) directly interact with PME and inhibit its activity, affecting pectin composition in the cell wall. Lionetti et al. [57] reported that overexpressing Arabidopsis *PMEI* increased the degree of pectin methylesterification by approximately 16%, resulting in longer roots due to the promotion of cell elongation. Therefore, the degree of methylation and demethylation of pectin determines the balance between extensibility and rigidity, affecting growth and cell shape. In tomato, *PMEU1*, a ubiquitously expressed pectin methylesterase gene, is expressed during early fruit development [58]. Terao et al. [59] recently reported the occurrence of rapid pectin metabolism during the early stage of fruit development in tomato: immunolocalization analysis demonstrated that methyl-esterified pectin levels in the ovary increased from 1 DBF to 3 DAF [59]. During fruit set, the transition of cell state from cell division to cell expansion occurs during a short period of time, and the regulation of this process is important for determining the size of the fruit. Therefore, it would be interesting to investigate whether PMEI plays a role in the post-translational regulation of PME and cell wall state during fruit set.

In addition, the pectinesterase inhibitor protein family includes several enzyme inhibitors such as invertase (Beta-fructofuranosidase) inhibitors, each of which has a specific target [60,61]. *Solyc09g011290* was annotated as an invertase inhibitor homolog in the SGN database. Invertase inhibitors regulate specific invertases in a post-translational manner, negatively affecting the enzyme activity of their targets [49,61]. We found that *Solyc09g011290* was specifically and highly expressed in the ovule/seed (S3 Table). The expression of *Solyc09g011290* was induced during anthesis and remained at high levels in the absence of pollination but was down-regulated by pollination and hormone treatments (S6 Fig). Several studies on the cell wall invertase (CWIN) and INVINH1 in tomato suggest that these proteins play important roles in seed set and fruit set by regulating the unloading of sugar from the phloem during the ovary-to-fruit transition [4,49,62,63]. Thus, the expression pattern of *Solyc09g011290*, the up-regulation during flowering and the down-regulation by the fruit-set stimulus (S6 Fig), suggests that *Solyc09g011290* may also participate in the modulation of the sugar unloading to unpollinated pistil via post-translational inhibition of invertase activity.

### Identification of pistil-specific transcriptional regulators

#### Pistil-specific transcription factors

Next, we performed similar RT-PCR analyses of several transcription factor genes listed among the PSGs and confirmed the tissue-specific expression of three transcription factor genes (Fig 3B).

*Solyc04g074320* (SlTT1-like) shares high homology (71.5%) with Arabidopsis zinc-finger protein TRANPARENT TESTA1 (TT1) [64]. Arabidopsis *TT1* expression is restricted to developing ovules and young seeds and functions in the accumulation of proanthocyanidin pigments in the seed coat [65,66], while in the current study, tomato *SlTT1-like* transcripts were exclusively detected in the ovule, embryo, and endosperm but not in the seed coat (Fig 3B). Mazzucato *et al*. [67] provided evidence that higher anthocyanin content is associated with increased early fruit growth in non-pollinated flowers. Furthermore, there is an evidence that the alteration of the flavonoid pathway via the regulation of biosynthesis genes induces seedless fruit development in both a pollination-dependent and pollination-independent manner [68,69]. Further elucidation of the function of *SlTT1-like* in the control of flavonoid-related genes may provide insight into the role of flavonoids during fruit set initiation.

*SlINO* (*Solyc05g005240*) was identified as a pistil-specific *YABBY* transcription factor gene (S2B Fig and Fig 3B). YABBY family proteins contain two conserved domains, i.e., a C2C2 zinc-finger-like domain in their N-termini and a helix-loop-helix domain known as the YABBY domain [70]. In Arabidopsis, two YABBY genes, *INO* and *CRC*, show tissue-specific expression in the pistil and are involved in pistil and early fruit development [44–46]. Nine YABBY genes were previously identified in tomato, three of which (*SlCRCa*, *Solyc01g0101240*; *SlCRCb*, *Solyc05g012050*; *SlINO*, *Solyc05g005240*) are specifically expressed in the flower bud and in open flowers at anthesis [50]. In the current study, we found that *SlCRCa*, *SlCRCb*, and *SlINO* were preferentially expressed in the pistil (S2B Fig). *SlCRCa* was expressed in the early stage of pistil development, while *SlCRCb* and *SlINO* were expressed during all stage of pistil development. Furthermore, we confirmed the tissue-specific expression of *SlINO* in pistils by RT-PCR analysis (Fig 3B), suggests its role in the regulation of pistil development [46].

*Solyc01g010600* (*SlATHB13/23-like*), which encodes a homeodomain leucine zipper 1 transcription factor (HD-Zip TF), shares similarity with *Arabidopsis* class-1 HD-Zip genes *AtHB13* and *AtHB23* and was specifically expressed in the pistil before anthesis (Fig 3B). The HD-Zip TF family forms a large gene family that is divided into four classes; 58 HD-Zip proteins found in both Arabidopsis and tomato are listed in PlantTFDB version 3.0 (http://planttfdb.cbi.pku.edu.cn) [71,72]. Although the molecular functions of class-1 HD-Zip proteins in the regulation of pistil development remain elusive, AtHB13 and AtHB23 were shown to play negative roles in inflorescence stem elongation by affecting cell division, and AtHB13 also regulates pollen hydration and development [73]. In tomato, class-1 HD-Zip SlHZ24 functions as a transcriptional activator of *SlGMP3* (encoding GDP-_D_-mannosepyrophosphorylase), which plays an important role in the production of the antioxidant ascorbate [74]. In addition, virus-induced gene silencing of class-1 HD-Zip *LeHB1* reduced the mRNA accumulation of *LeACO1* and inhibited ripening [75]. Further, Lin et al. [75] also reported that ectopic overexpression of *LeHB1* led to the conversion of sepals into carpel-like structures. We also found that *SlATHB13/23-like* was highly expressed in the ovary wall in the pistil at anthesis (S3 and S4 Tables), suggesting its regulatory role in carpel and ovary wall development.

Besides, we identified the type-I MADS box gene, *Solyc01g106730* among PSGs (Table 1). MADS box genes encode transcription factors which are generally involved in homeotic regulation of reproductive organs and can be divided into two subfamilies (type-I and type-II) according to the presence of conserved domains [75, 76]. Some type-II MIKC MADS box genes play key roles as regulators of meristem identity, flowering time, and fruit and seed development [77,78], whereas little information is available about the function of type-I MADS box proteins. Most (38 out of 61) Arabidopsis type-I MADS box genes are expressed in the female gametophyte [79], whereas others exhibit highly specific expression such as in the central cell and embryo sac [80]. For example, Arabidopsis type-I *AGAMOUS-LIKE61*(*AGL61*)*/DIANA* (*DIA*) is expressed exclusively in the central cell and early endosperm and plays crucial role in endosperm development after fertilization through transcriptional control in the central cells [79–82]. *AtAGL62* also plays important role in the endosperm and seed coat development [83–85]. Gene expression analysis using tissue-specific transcriptome data from wild tomato *S*. *pimpinellifolium* [27] revealed that the type-I MADS box gene *Solyc01g106730* is preferentially expressed in the ovule (S3 and S4 Tables). In addition to the relationship between type-I MADS box genes and seed development, there is an evidence that down-regulation or mutation in type-II MADS box genes, such as *TM29*, *TAP3*, *TM8*, *SlAGL11* or *SlAGL6*, results in parthenocarpy [86–90]. Thus, it would be important to investigate the roles of *Solyc01g106730* in pistil, seed, and fruit development.

#### Pistil-specific peptide hormone-like small peptide genes and receptor-like proteins

The role of peptide hormones in plant signaling pathways is a popular focus of study [91–95]. The peptide hormone signaling system involves two main components: (1) small ligand proteins such as small cysteine-rich peptides (CRPs) and (2) receptor proteins such a leucine-rich receptor-like kinases (LRR-RLKs) [96]. CRPs function as signaling molecules (peptide hormones) in various plant species, which are required for many aspects of development including antimicrobial defense, pollen tube guidance, stomatal patterning, and early embryo patterning [97–104]. CRPs contain four, six, or eight conserved cysteine residues at their C-termini in addition to a secretion signal at their N-termini. Interestingly, a substantial number of PSGs identified in this study encode small proteins (44 out of 108 genes identified by the mapping-based method [40%]) less than 200 amino acids in length (Table 2). Small proteins are defined as proteins smaller than 200 amino acids according to previous reports [94,97,105]. Since peptide hormone-like small proteins share a conserved structure, we performed a sequence similarity search of the 44 identified small proteins and one TAPETUM DETERMINANT 1 (TPD1)-like protein (204 aa) by BLAST analysis and SignalP 4.1 server (http://www.cbs.dtu.dk/services/SignalP/) manually to investigate whether they have conserved residues or functional domains. Roughly half of these proteins also have a secretion signal in their N-termini (Table 2). Notably, through subsequent sequence analysis of these small proteins, four tissue-specific CRPs including an unknown gene (*Solyc06g075200*) and two LRR-RLK-like proteins were identified (Listed in Table 2, S5 Fig).

**Table 2:** List of pistil-specific or preferentially expressed small proteins.

#	ITAG ID	Description in ITAG2.40	length (aa)	*Presence of predicted secreted signal (aa)	Homologue in Arabidopsis	length (aa)	Identities (%)		Description	"Expression in pistil of Moneymaker (Ovule and/or ovary wall) from Zhang et al (2016)^[^^23^^]^"
PSSP1	Solyc01g016530	Unknown Protein (AHRD V1); contains Interpro domain(s) IPR008507 Protein of unknown function DUF789	87	-	AT1G73210	314	32/69	46	Protein of unknown function (DUF789)	Ovule
PSSP2	Solyc01g081360	Unknown Protein (AHRD V1)	151	1–29	-	-	-	-	-	Ovule
PSSP3	Solyc01g108380	Protease inhibitor protein (AHRD V1 -**- B3FNP9_HEVBR); contains Interpro domain(s) IPR000864 Proteinase inhibitor I13, potato inhibitor I	77	-	AT2G38900	88	27/61	44	Serine protease inhibitor, potato inhibitor I-type family protein	Ovule
PSSP4	Solyc02g069330	Unknown Protein (AHRD V1); contains Interpro domain(s) IPR006501 Pectinesterase inhibitor	180	1–19	AT5G64620	180	26/80	33	C/VIF2, ATC/VIF2	Ovule
PSSP5	Solyc03g058330	Unknown Protein (AHRD V1)	108	-	AT5G06760	158	57/144	40	LEA4-5	Ovule
PSSP6	Solyc04g081180	Unknown Protein (AHRD V1)	79	-	-	-	-	-	-	Ovule
PSSP7	Solyc05g010200	Unknown Protein (AHRD V1)	115	1–25	-	-	-	-	-	Ovule
PSSP8	Solyc06g048400	Unknown Protein (AHRD V1); contains Interpro domain(s) IPR008502 Protein of unknown function DUF784, Arabidopsis thaliana	155	-	AT3G30387	115	34/97	35	Protein of unknown function (DUF784)	Ovule
PSSP9	Solyc06g075200	Unknown Protein (AHRD V1)	81	1–22	AT5G37474	80	28/83	34	Putative membrane lipoprotein	Ovule
PSSP10	Solyc07g062320	Unknown Protein (AHRD V1)	79	-	-	-	-	-	-	Ovule
PSSP11	Solyc08g080020	Serine protease inhibitor potato inhibitor I-type family protein (AHRD V1 ***- D7LT19_ARALY); contains Interpro domain(s) IPR000864 Proteinase inhibitor I13, potato inhibitor I	104	1–19	AT3G46860	85	32/86	37	Serine protease inhibitor, potato inhibitor I-type family protein	Ovule
PSSP12	Solyc09g011280	Unknown Protein (AHRD V1); contains Interpro domain(s) IPR006501 Pectinesterase inhibitor	178	1–23	AT3G17220	173	31/131	24	ATPMEI2	Ovule
PSSP13	Solyc09g089590	Ramosa1 C2H2 zinc-finger transcription factor (AHRD V1 *-*- D0UTY8_ZEAMM); contains Interpro domain(s) IPR007087 Zinc finger, C2H2-type	197	-	AT3G23130	204	78/192	78	SUP, FON1, FLO10	Ovule
PSSP14	Solyc11g005500	ECA1 protein (AHRD V1 *-*- Q53JF8_ORYSJ); contains Interpro domain(s) IPR010701 Protein of unknown function DUF1278	130	1–26	AT1G76750	158	63/124	51	EC1.1	Ovule
PSSP15	Solyc11g005540	ECA1 protein (AHRD V1 *-*- Q53JF8_ORYSJ); contains Interpro domain(s) IPR010701 Protein of unknown function DUF1278	136	1–16	AT2G21750	125	61/130	47	EC1.3	Ovule
PSSP16	Solyc11g006840	Unknown Protein (AHRD V1)	126	-	-	-	-	-	-	Ovule
PSSP17	Solyc09g025200	Ribosomal protein L18 (AHRD V1 *-*- B7FMF5_MEDTR); contains Interpro domain(s) IPR000039 Ribosomal protein L18e	72	1–22	AT3G05590	187	31/50	62	RPL18	Ovary wall
PSSP18	Solyc09g056030	Unknown Protein (AHRD V1)	82	-	AT4G12570	873	17/44	39	UPL5	Ovary wall
PSSP19	Solyc01g007270	Cytokinin riboside 5&apos;-monophosphate phosphoribohydrolase LOG (AHRD V1 **—LOG_ORYSJ)	70	-	AT5G06300	217	56/68	82	-	Not detected
PSSP20	Solyc01g079560	B3 domain-containing protein Os11g0197600 (AHRD V1 ***- Y1176_ORYSJ); contains Interpro domain(s) IPR003340 Transcriptional factor B3	109	-	AT3G18990	341	30/92	33	VRN1, REM39	Not detected
PSSP21	Solyc02g032150	Unknown Protein (AHRD V1)	147	-	-	-	-	-	-	Not detected
PSSP22	Solyc02g084140	Unknown Protein (AHRD V1)	132	-	-	-	-	-	-	Not detected
PSSP23	Solyc03g116410	Zinc finger CCCH domain-containing protein 39 (AHRD V1 ***- C3H39_ARATH); contains Interpro domain(s) IPR000571 Zinc finger, CCCH-type	117	-	AT3G19360	386	54/199	27	Zinc finger (CCCH-type) family protein	Not detected
PSSP24	Solyc04g025740	Homeobox-leucine zipper protein ROC3 (AHRD V1 ***- ROC3_ORYSJ); contains Interpro domain(s) IPR001356 Homeobox	148	-	AT1G73360	722	52/125	42	HDG11, EDT1, ATHDG11	Not detected
PSSP25	Solyc04g051070	Unknown Protein (AHRD V1)	80	-	-	-	-	-	-	Not detected
PSSP26	Solyc04g078240	Natural resistance associated macrophage protein (AHRD V1 *-—B3W4E1_BRAJU); contains Interpro domain(s) IPR001046 Natural resistance-associated macrophage protein	161	-	AT1G47240	530	73/95	77	NRAMP2, ATNRAMP2	Not detected
PSSP27	Solyc05g013230	Unknown Protein (AHRD V1)	118	-	AT3G23880	364	21/57	37	F-box and associated interaction domains-containing protein	Not detected
PSSP28	Solyc07g054360	Unknown Protein (AHRD V1)	142	-	-	-	-	-	-	Not detected
PSSP29	Solyc08g061120	Unknown Protein (AHRD V1)	190	-	-	-	-	-	-	Not detected
PSSP30	Solyc09g073020	Unknown Protein (AHRD V1)	50	-	-	-	-	-	-	Not detected
PSSP31	Solyc09g075110	Unknown Protein (AHRD V1)	63	-	-	-	-	-	-	Not detected
PSSP32	Solyc10g047720	Unknown Protein (AHRD V1)	172	-	AT5G26805	156	44/163	27	unknown protein	Not detected
PSSP33	Solyc10g055600	S-phase kinase-associated protein 1A (AHRD V1 **—B2VUU5_PYRTR); contains Interpro domain(s) IPR001232 SKP1 component	51	-	AT4G34210	152	38/47	81	ASK11, SK11	Not detected
PSSP34	Solyc01g104390	Blue copper protein (AHRD V1 **—B6TT37_MAIZE); contains Interpro domain(s) IPR003245 Plastocyanin-like	122	1–27	AT1G17800	129	49/116	42	ARPN	Both
PSSP35	Solyc02g078090	Unknown Protein (AHRD V1)	105	1–26	-	-	-	-	-	Both
PSSP36	Solyc03g123770	Unknown Protein (AHRD V1)	112	-	-	-	-	-	-	Both
PSSP37	Solyc03g123970	Lipid-binding serum glycoprotein family protein (AHRD V1 *-*- D7LAX8_ARALY)	116	1–17	AT3G20270	722	26/51	51	lipid-binding serum glycoprotein family	Both
PSSP38	Solyc04g014750	TNFR/CD27/30/40/95 cysteine-rich region (AHRD V1 ***- Q2HT38_MEDTR)	105	1–32	AT1G12064	109	34/73	47	Unkown protein	Both
PSSP39	Solyc05g005240	YABBY-like transcription factor CRABS CLAW-like protein (AHRD V1 **-* Q6SRZ7_ANTMA); contains Interpro domain(s) IPR006780 YABBY protein	192	-	AT1G23420	231	100/184	54	INO	Both
PSSP40	Solyc05g010190	Unknown Protein (AHRD V1)	138	1–23	AT3G42565	119	48/121	40	ECA1 gametogenesis related family protein	Both
PSSP41	Solyc07g032700	Unknown Protein (AHRD V1)	120	-	-	-	-	-	-	Both
PSSP42	Solyc07g053400	Unknown Protein (AHRD V1)	97	-	-	-	-	-	-	Both
PSSP43	Solyc09g011290	Invertase inhibitor homolog (AHRD V1 ***- O49603_ARATH); contains Interpro domain(s) IPR006501 Pectinesterase inhibitor	188	1–24	AT5G64620	180	52/173	30	C/VIF2, ATC/VIF2	Both
PSSP44	Solyc09g091300	Self-incompatibility protein (Fragment) (AHRD V1 -**- C8C1B5_9MAGN); contains Interpro domain(s) IPR010264 Plant self-incompatibility S1	148	1–23	AT3G26880	161	35/135	33	Plant self-incompatibility protein S1 family	Both
PSSP45	Solyc11g012650	TPD1 (AHRD V1 *-*- Q6TLJ2_ARATH)	204	1–28	AT1G32583	179	66/112	59	TPD1-like	Ovule

* Presence of secreted signal sequence in each protein was predicted by SignalP 4.1 Server with default setting.

*OPE4* (*Solyc11g012650*) was homologous to Arabidopsis *TAPETUM DETERMINANT* (*AtTPD1*, AT4G24972) (6e-37), encoding a peptide hormone that functions as a ligand molecule to regulate the specification of tapetum cells in coordination with receptor protein EMS/EXS [106, 107], while BLAST searches of the tomato genome identified three other homologs, designated *SlTPD1L1* (*Solyc03g097530*), *SlTPD1L2/OPE4* (*Solyc11g012650*), and *SlTPD1L3* (*Solyc11g006850*), based on sequence similarity to *AtTPD1*, with 59.4% (1e-49), 55% (6e-37), and 50% (4e-33) sequence similarity, respectively. Like AtTPD1, we confirmed the presence of a secretion signal in the N-terminal region and conserved cysteine residues at the C-terminus among the three deduced proteins (S3A Fig). Although the sequence of N-terminal secretion signal region varied among the three proteins, an alignment of each SlTPD1L compared to amino acids 26–179 of AtTPD1 revealed a high degree of similarity (48–56%) (S3B Fig). Although it is known that *AtTPD1* is also expressed in inflorescence meristems, floral meristems, carpel primordia, and ovule primordia, its function in these tissues remains unknown [106, 107]. The notion that *SlTPD1L2/OPE4* (*Solyc11g012650*) showed pistil-specific expression (Fig 3B), and that *OPE4* is shown to be preferentially expressed in the ovule both in *S*. *pimpinellifolium* and tomato cultivar ‘Moneymaker’ (S3 and S4 Tables), it was suggested that *SlTPD1L2/OPE4* might play a tissue-specific role in ovule development.

*Solyc11g005540*, *Solyc11g005500*, and *Solyc05g010190* share sequence similarity with Arabidopsis ECA1-like protein (EC1) genes, which involved in gamete fusion [108] (S4B and S4C Fig). mRNA from the five Arabidopsis *EC1* genes (*EC1*.*1* to *EC1*.*5*; belonging to the ECA1 [Early Culture Abundant] gametogenesis-related cysteine-rich protein subfamily) is present only in egg cells before fertilization. And, small proteins encoded by *EC1* genes are secreted into the outer region of the egg cell to redundantly regulate the fusion of the germ cell to the sperm cell during double fertilization [82,108]. Three tomato EC1-like protein homologs are expressed in the ovule/seed in *S*. *pimpinellifolium* (S3 and S4 Tables), while in the current study, we confirmed the pistil-specific expression of *Solyc11g005500* and *Solyc11g005540* (S4A Fig). Sequence similarity analysis of these protein sequences with AtEC1s identified five tomato homologs, which share six conserved cysteine residues in the C-terminal region and an N-terminal secretion signal peptide (S4B Fig). Therefore, these genes were designated *S*. *lycopersicum* SlEC1-like genes (SlECLs; e.g., *SlECL1* [*Solyc11g005500*], *SlECL3* [*Solyc05g010190*], and *SlECL5* [*Solyc11g005540*]), suggesting that they play a conserved role in the pistil at fertilization.

In addition to several ligand-like, pistil-specific CRPs, we also identified two proteins with RLK-related domain; *OPE5* (*Solyc10g051370*) and *Solyc03g096190*. *OPE5* was expressed in ovule and seed, and *Solyc03g096190* was expressed in placenta and septum (S3 Table). In tomato, the LRR-RLK family represents the largest family of RLKs [109]. Molecular genetic studies have shown that LRR-RLKs are involved in a wide range of plant development, such as stem cell maintenance [110,111], cell fate determination and patterning [103,112], and brassinosteroid signaling [113–115]. Generally, LRR-RLK proteins, such as EMS1/EXS and CLAVATA1 (CLV1), function as receptors by interacting with small ligand proteins at LRR domain and subsequently transmit external signals into cells by activating kinase domain, inducing various cellular responses [116–118]. While *Solyc03g096190* possesses the LRR domain, one transmembrane domain and the cytoplasmic kinase domain, *OPE5* possesses only small LRR domain composed of five repeats (S5 Fig). Some receptor-like proteins with extracellular LRR domains lacking the internal kinase domain also have been identified in other plants, such as *Arabidopsis* CLAVATA2 (CLV2) [119], which regulates various developmental and immunity signaling pathways by interacting with other RLKs including CLV1 [119–122]. Unraveling the role of pistil-specific RLK like proteins may help understand the detailed mechanism of signal transduction during fruit set.

### Relationship between PSGs and fruit set signaling

We further characterized and identified PSGs associated with fruit set in the pistil. Specifically, we investigated their expression in a dataset of differentially expressed genes (DEGs) in the pistils of plants treated with 2,4-D and GA_3_ from Tang et al. [20]. Among total 4764 and 6875 DEGs in ovaries undergoing fruit set after induction by plant hormones (auxin and GA) or pollination at 4 DAF compared to unpollinated ovaries at 2 DBF and unpollinated ovaries at 4 DAF, respectively, three genes, including *invertase inhibitor homolog* (*Solyc09g011290*), *SlATHB13/23-like* (*Solyc01g010600*), *SlCKX8* (*Solyc10g017990*) were included out of 108 PSGs, suggesting their roles in pistil development and/or fruit set initiation (S6 Fig). The mRNA levels of *SlATHB13/23-like* (*Solyc01g010600*) were significantly reduced at 4 DAF by either plant hormone (auxin or GA) treatment or pollination compared to that at 2 DBF, while no significant difference was found compared to ovaries at 6-days post-emasculation (6 DPE). By contrast, the mRNA levels of *SlCKX8* (*Solyc10g017990*), *invertase inhibitor homolog* (*Solyc09g011290*) were significantly lower in ovaries undergoing fruit set compared to those at 6 DPE, indicating that these genes are up-regulated in the absence of fruit set signaling after flowering.

### Regulatory regions of PSGs as genetic engineering tools

Tissue-specific promoters are useful tools for regulating the expression of genes of interest in a spatial and temporal manner, which could provide new insights into various biological mechanisms and facilitate their application to molecular breeding, such as generating genetically modified organisms [123]. Constitutive promoters such as the 35S promoter from *Cauliflower mosaic virus* (CaMV) is widely used to regulate the expression of target genes in plants [124,125]. However, the constitutive regulation of target genes is not always useful, since it sometimes induces additional undesirable effects that hamper agronomic applications. Thus, in the past several decades, many tissue-specific promoters have been isolated, such as fruit-specific promoters [126–129], root-specific promoters [130–132], seed-specific promoters [133–135], many pollen and/or anther-specific promoters [36,136–139], and so on. Several individual promoters targeting the stigma and/or style in the pistil have also been evaluated in several plant species, such as the stigma- and style-specific thaumatin/PR5-like protein (*PsTL1*) promoter from Japanese pear (*Pyrus serotina*) [140–142]. Importantly, the use of the ovule-specific *DefH9* and *INO* promoters allows parthenocarpy to be efficiently induced via the ovule-specific activation of auxin signaling without producing substantial undesirable fruit traits in tomato [143]. Moreover, several reproductive tissue-specific promoters, such as sperm cell- and egg cell-specific promoters, have been successfully used to induce targeted mutagenesis by CRISPR/Cas9 [36,144]. For example, Wang et al. [144] used the promoter of egg cell-specific *EC1*.*2* (homolog of *SlEC1*.*2* and *SlEC1*.*3* identified in the current study) for CRISPR/Cas9 to generate homozygous mutants for multiple target genes in a single generation in Arabidopsis. These findings indicate that pistil-specific promoters, especially ovule-specific promoters, can be highly effective tools for plant breeding. Here, we identified various types of PSGs in tomato, e.g., *SlINO* and two homologs of Arabidopsis *EC1*.*2*, which were specifically expressed in the ovule (Table 2). Thus, these promoters could contribute to tissue-specific regulation or genome editing of target genes to improve fruit set by inducing parthenocarpy.

## Conclusion

**Fig 4:**
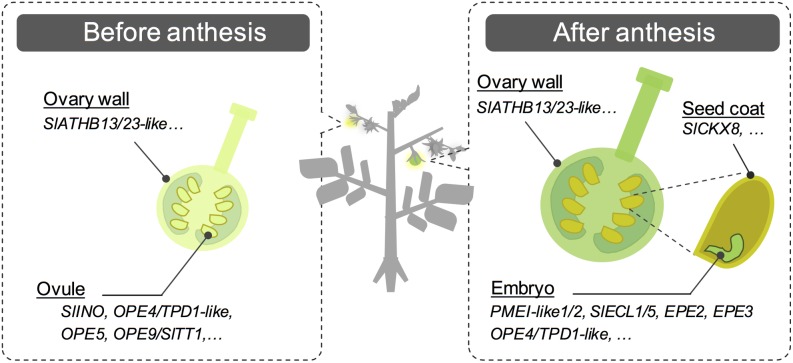
PSGs identified from this study. Many candidate transcriptional regulators in tomato pistils were identified, including cysteine-rich peptide (CRP)-like proteins, indicating their roles in the development of specific types of cells in the pistil. Ovule- and embryo-specific genes: *SlINO*, *SlTT1-like*, *SlECL1*, *SlECL5*. Pericarp-specific gene; *SlATHB13/23-like*. Seed coat-specific gene; *SlCKX8*.

In conclusion, in this study, we conducted global analysis of tomato PSGs in tomato, which might be involved in the ovary development or fruit set process, by performing RNA-seq analysis and comparisons with publically available data. This study successfully identified several genes encoding signaling-related transcription factor and peptide hormone-like proteins, in addition to many genes with unknown functions (Fig 4). Although their biological functions remain to be determined, our findings lay the foundation for further analysis of the precise gene regulatory network and developmental mechanisms underlying fruit set, in addition to the usage of promoter region of PSGs for genetic engineering and molecular breeding.

## Supporting information

S1 Fig Results of trimming and filtering of raw read data from each sample obtained by RNA-seq. Trimming was performed using FastQC. #1 and #2 represent FastQC analysis of original and trimmed data from petals, respectively. Pistil and fruit samples (#3–10): pistils of 2–2.5 mm buds (#3), 3–4 mm buds (#4), 1 DBF (#5), anthesis (#6), 5 DAF (#7), 5 mm ovaries at 7 DAF (#8), mature green fruits (#9), and red fruits (#10); Stamen and other floral organ samples (#12–14): stamens of 3–4 mm buds (#12), 1 DBF (#13) and anthesis (#14). sepals; Vegetative organs (#15–18): 3-week-old leaves (#15), mature leaves (#16), stems (#17), roots (#18), from left to right, respectively.
(TIF)

S2 Fig Identification of pistil-specific genes based on the direct-mapping method. (A) Number of expressed genes in different tissue/stages. Genes with RPKM values greater than 0 and 0.5 are shown in the top and bottom panels, respectively. (B) Expression of tomato YABBY transcription factor family genes. The expression of nine YABBY transcription factor genes was examined. *SlCRCa*, *SlCRCb*, and *SlINO* appeared to be preferentially expressed in the pistil. Vertical axis represents the expression value (RPKM). Horizontal axis represents the 17 samples used for RNA-seq analysis.
(TIF)

S3 Fig TPD1-like cysteine-rich peptides specifically expressed in pistils. (A) Alignment of five TPD1-like proteins in tomato, including SlTPD1 (Solyc11g005500), SlTPD1-like1 (Solyc12g009850), TPD1-like2 (Solyc05g010190), TPD1-like3 (Solyc04g071640), and TPD1 (AT4G24972). rice MIL2/TPD1A (Os12g0472500), maize MAC1 (JN247438). (B) Phylogenetic tree of three tomato TPD1-like proteins and several orthologs of Arabidopsis, rice and maize. Numbers above the branches indicate bootstrap values (10,000 replicates).
(TIF)

S4 Fig EC1-like cysteine-rich peptides specifically expressed in pistils. (A) Expression of tomato EC1-like (ECL) genes. Both *SlECL1* and *SlECL5* were specifically expressed in the pistil at anthesis. Bottom one represents the expression of the internal control gene SAND [41]. (B) Alignment of five ECA1-like proteins in tomato, including SlECL1 (Solyc11g005500), SlECL2 (Solyc05g010190), SlECL3 (Solyc12g009850), SlECL4 (Solyc04g071640), and SlECL5 (Solyc11g005540). Arabidopsis EC1s and Tomato ECLs share six conserved cysteine residues at their C-termini (asterisk). (C) Neighbor-joining tree of amino acid sequences of five tomato ECLs proteins and five Arabidopsis EC1s. Numbers above the branches indicate bootstrap values (10,000 replicates).
(TIF)

S5 Fig Second structure of CRPs and LRR-RLKs identified in this study. Conserved domains and motifs were searched using CDD in NCBI. The presence of secretion signal and transmembrane region was investigated using SignalP 4.1 Server and TMHMM Server v. 2.0 (http://www.cbs.dtu.dk/services/TMHMM/).
(TIF)

S6 Fig Five *PSGs* that are differentially regulated during fruit set. The data were obtained from Tang et al. 2015. Vertical axis represents the expression values normalized to transcripts per million (TPM) 6. Horizontal axis represents the pistil sample types.
(TIF)

S1 Table Tomato tissues subject to RNA-seq and summary of sequencing results. (XLSX)

S2 Table Gene expression patterns of the 108 genes in various tissues of Micro-Tom. (XLSX)

S3 Table Gene expression patterns of the 108 PSGs in the pistils of wild tomato relative *S*. *pimpinellifolium* based on published data from Pattison et al. (2015). (XLSX)

S4 Table Gene expression patterns of the 108 PSGs based on published data from Zhang et al. (2016). (XLSX)

S5 Table List of oligonucleotide primers used for RT-PCR in this study. List of ovule preferentially expressed genes (*OPEs*) and embryo preferentially expressed genes (*EPEs*). (XLSX)
