# Supplementary figures and images for: Genome-wide identification of pistil-specific genes expressed during fruit set initiation in tomato (Solanum lycopersicum)
Source: PLoS One. 2017 Jul 6;12(7):e0180003. doi: 10.1371/journal.pone.0180003 (PMC5500324; doi:10.1371/journal.pone.0180003)

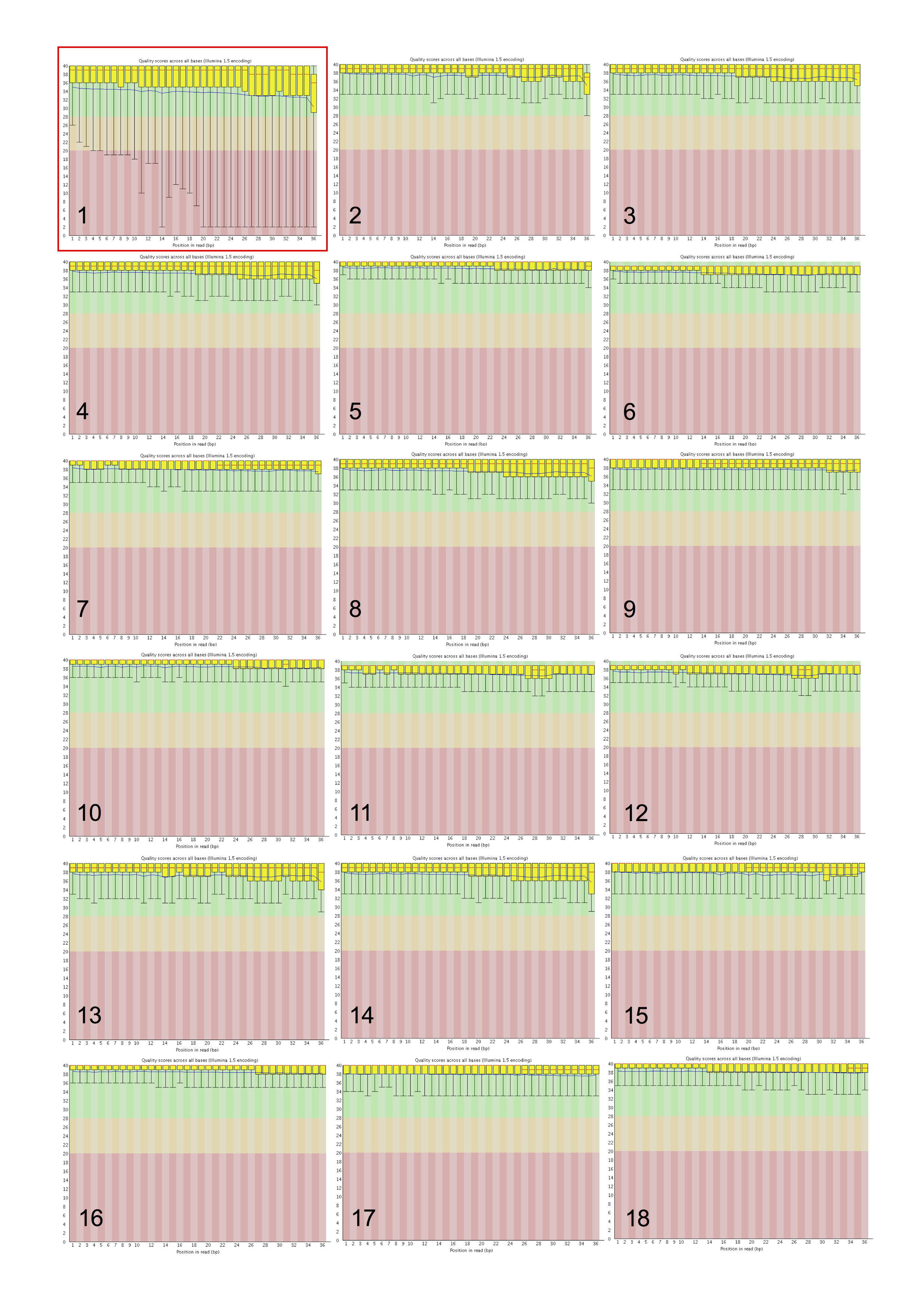

Supplement: S1 Fig — Trimming was performed using FastQC. #1 and #2 represent FastQC analysis of original and trimmed data from petals, respectively. Pistil and fruit samples (#3–10): pistils of 2–2.5 mm buds (#3), 3–4 mm buds (#4), 1 DBF (#5), anthesis (#6), 5 DAF (#7), 5 mm ovaries at 7 DAF (#8), mature green fruits (#9), and red fruits (#10); Stamen and other floral organ samples (#12–14): stamens of 3–4 mm buds (#12), 1 DBF (#13) and anthesis (#14). sepals; Vegetative organs (#15–18): 3-week-old leaves (#15), mature leaves (#16), stems (#17), roots (#18), from left to right, respectively. (TIF) [file pone.0180003.s001.tif]

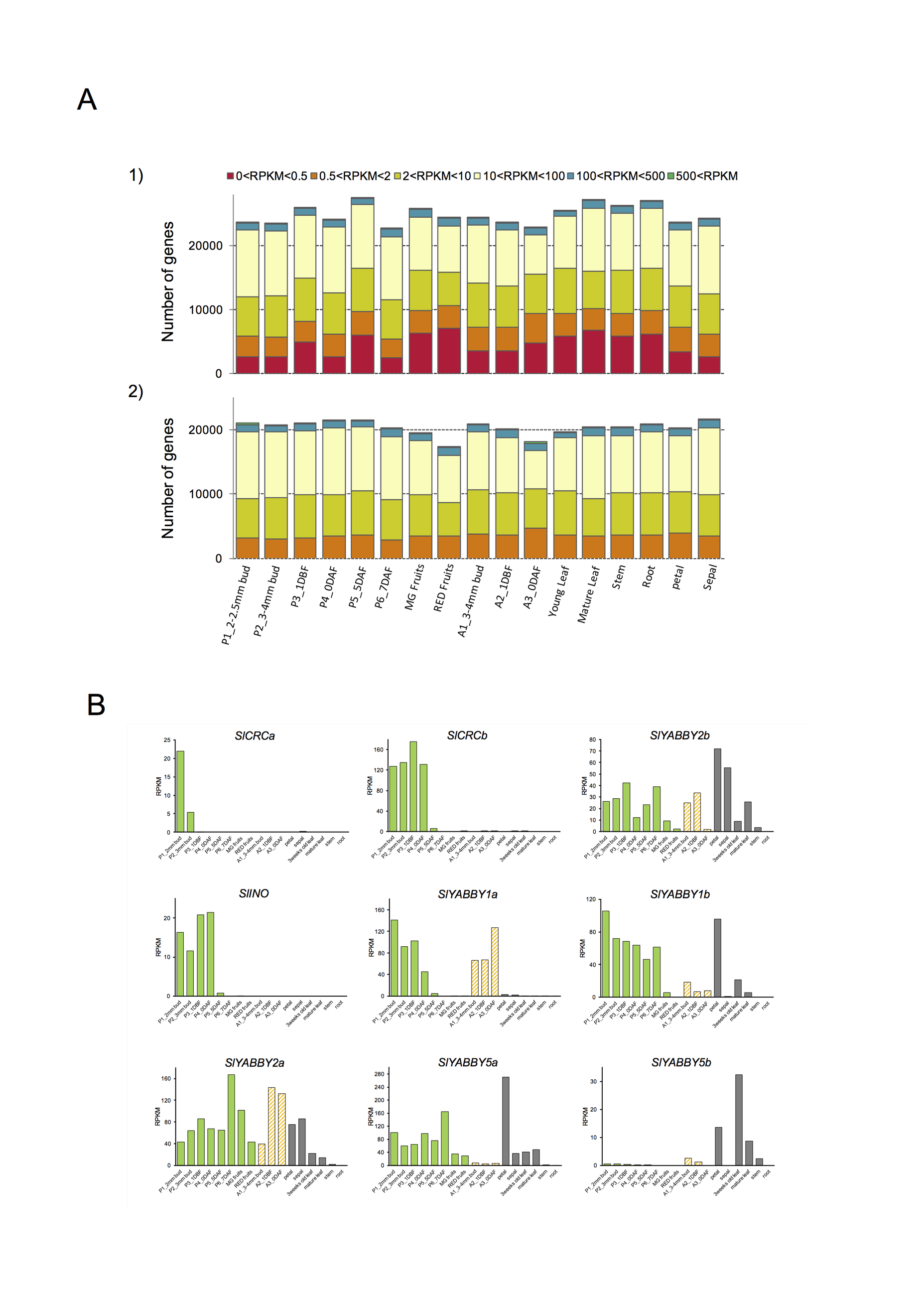

Supplement: S2 Fig — (A) Number of expressed genes in different tissue/stages. Genes with RPKM values greater than 0 and 0.5 are shown in the top and bottom panels, respectively. (B) Expression of tomato YABBY transcription factor family genes. The expression of nine YABBY transcription factor genes was examined. SlCRCa, SlCRCb, and SlINO appeared to be preferentially expressed in the pistil. Vertical axis represents the expression value (RPKM). Horizontal axis represents the 17 samples used for RNA-seq analysis. (TIF) [file pone.0180003.s002.tif]

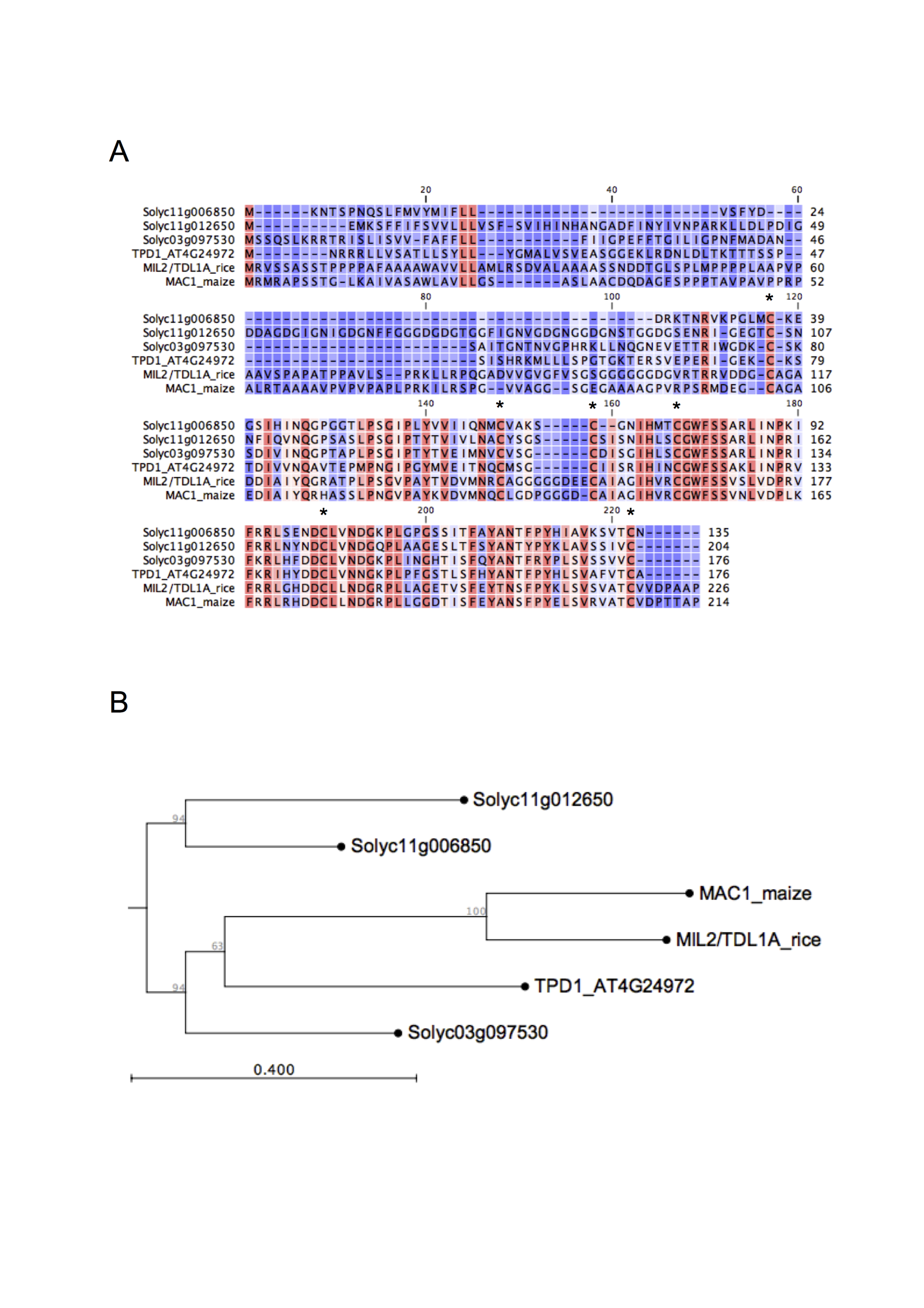

Supplement: S3 Fig — (A) Alignment of five TPD1-like proteins in tomato, including SlTPD1 (Solyc11g005500), SlTPD1-like1 (Solyc12g009850), TPD1-like2 (Solyc05g010190), TPD1-like3 (Solyc04g071640), and TPD1 (AT4G24972). rice MIL2/TPD1A (Os12g0472500), maize MAC1 (JN247438). (B) Phylogenetic tree of three tomato TPD1-like proteins and several orthologs of Arabidopsis, rice and maize. Numbers above the branches indicate bootstrap values (10,000 replicates). (TIF) [file pone.0180003.s003.tif]

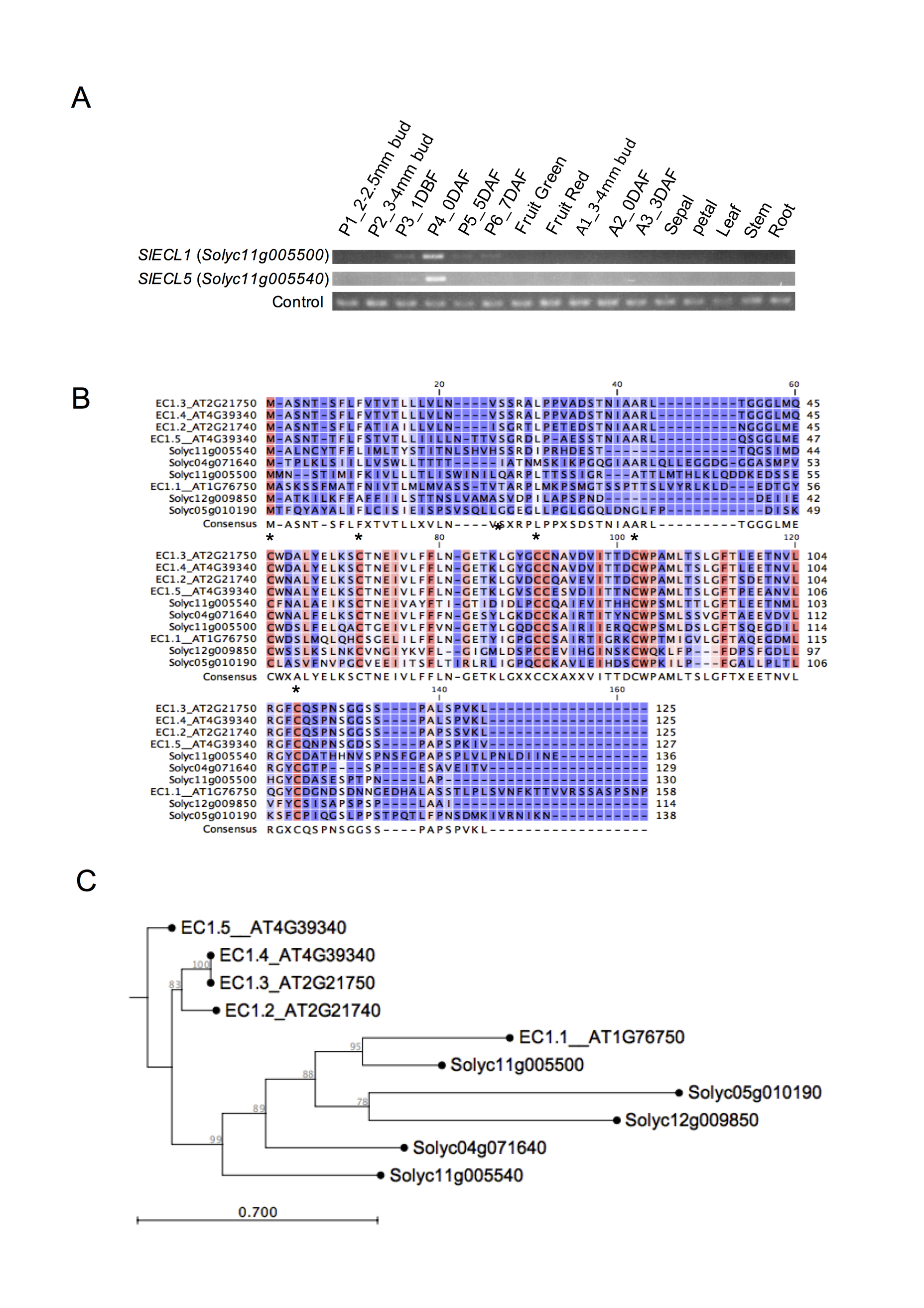

Supplement: S4 Fig — (A) Expression of tomato EC1-like (ECL) genes. Both SlECL1 and SlECL5 were specifically expressed in the pistil at anthesis. Bottom one represents the expression of the internal control gene SAND [41]. (B) Alignment of five ECA1-like proteins in tomato, including SlECL1 (Solyc11g005500), SlECL2 (Solyc05g010190), SlECL3 (Solyc12g009850), SlECL4 (Solyc04g071640), and SlECL5 (Solyc11g005540). Arabidopsis EC1s and Tomato ECLs share six conserved cysteine residues at their C-termini (asterisk). (C) Neighbor-joining tree of amino acid sequences of five tomato ECLs proteins and five Arabidopsis EC1s. Numbers above the branches indicate bootstrap values (10,000 replicates). (TIF) [file pone.0180003.s004.tif]

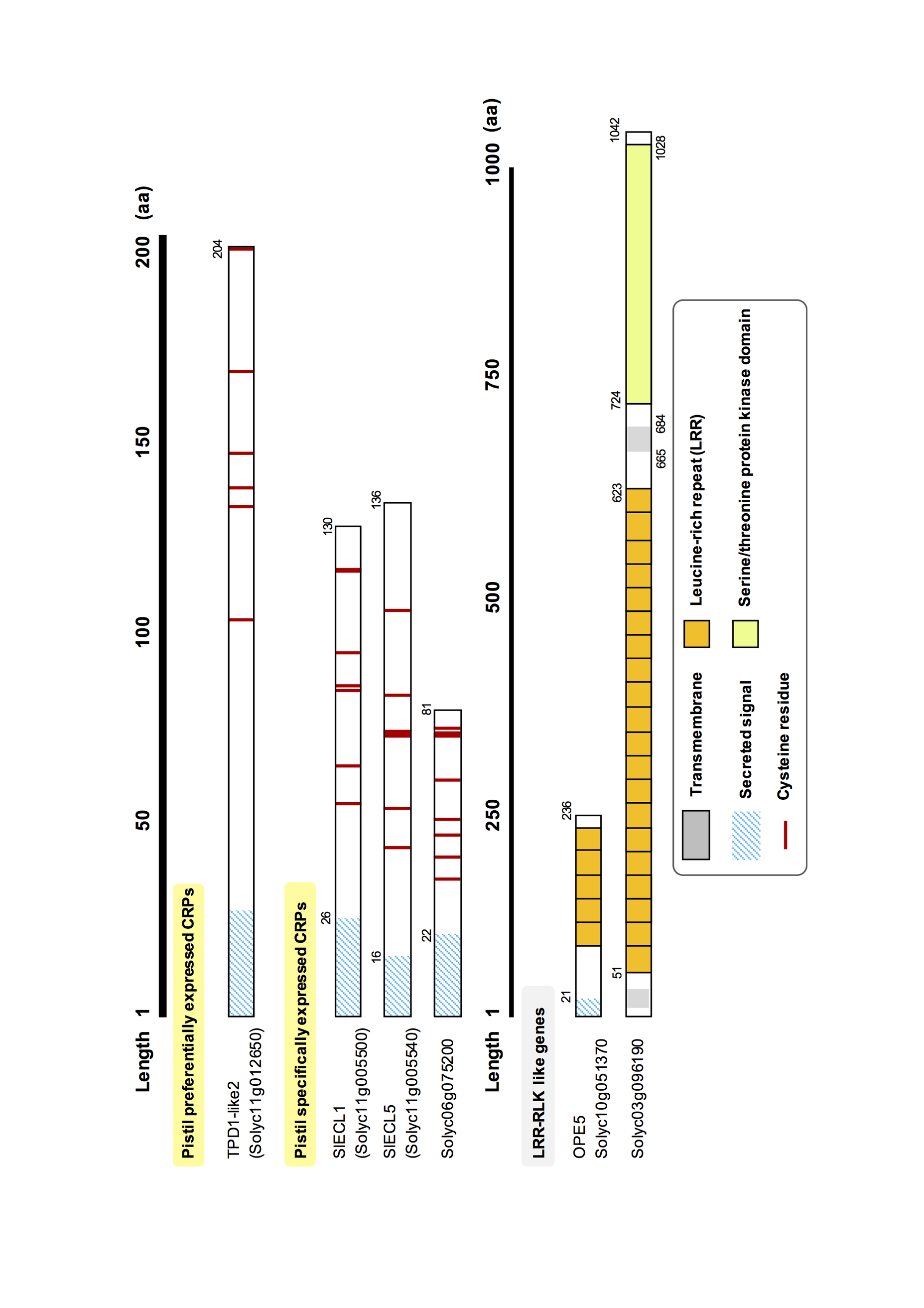

Supplement: S5 Fig — Conserved domains and motifs were searched using CDD in NCBI. The presence of secretion signal and transmembrane region was investigated using SignalP 4.1 Server and TMHMM Server v. 2.0 (http://www.cbs.dtu.dk/services/TMHMM/). (TIF) [file pone.0180003.s005.tif]

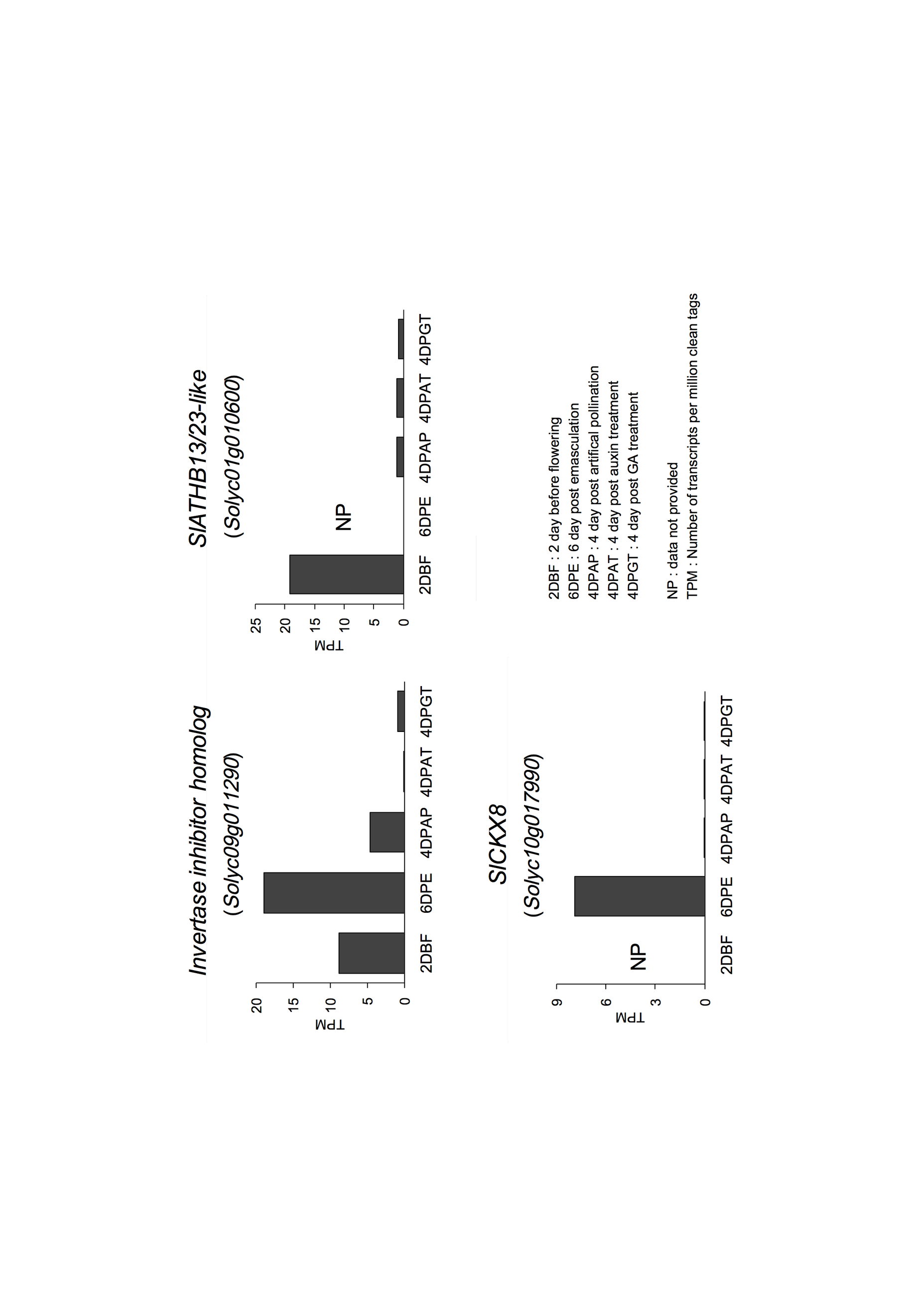

Supplement: S6 Fig — The data were obtained from Tang et al. 2015. Vertical axis represents the expression values normalized to transcripts per million (TPM) 6. Horizontal axis represents the pistil sample types. (TIF) [file pone.0180003.s006.tif]
